# Catalase-like metal–organic framework nanoparticles to enhance radiotherapy in hypoxic cancer and prevent cancer recurrence†
†Electronic supplementary information (ESI) available: Experimental details and supplementary figures. See DOI: 10.1039/c9sc00747d


**DOI:** 10.1039/c9sc00747d

**Published:** 2019-04-25

**Authors:** Yuanyuan Chen, Hui Zhong, Jianbo Wang, Xiuyan Wan, Yanhua Li, Wei Pan, Na Li, Bo Tang

**Affiliations:** a College of Chemistry, Chemical Engineering and Materials Science , Collaborative Innovation Center of Functionalized Probes for Chemical Imaging in Universities of Shandong , Key Laboratory of Molecular and Nano Probes , Ministry of Education , Institute of Molecular and Nano Science , Shandong Normal University , Jinan 250014 , P. R. China . Email: lina@sdnu.edu.cn ; Email: tangb@sdnu.edu.cn; b Radiation Department , Qilu Hospital of Shandong University , Jinan 250100 , P. R. China

## Abstract

Tumor hypoxia typically occurs inside a solid tumor with an inadequate oxygen supply, sharply reducing the therapeutic efficiency of radiotherapy and significantly increasing the risk of local tumor recurrence. Herein, we designed folic acid modified enzyme-like hafnium-based manganoporphyrin metal–organic framework nanoparticles (MnTCPP–Hf–FA MOF NPs) to overcome hypoxia-induced radioresistance and prevent postoperative recurrence. Hf, a high-Z element, can effectively absorb X-ray energy and convert O_2_ and H_2_O into reactive oxygen species to induce cell apoptosis. The MnTCPP ligand has an enzyme-like ability to catalytically decompose endogenous H_2_O_2_ into O_2_ for enhancing RT in hypoxic tumors. *In vivo* experiments revealed that the MOF NPs could effectively inhibit melanoma growth and prevent tumor postoperative recurrence with only one X-ray irradiation after intravenous injection. We expect that the current study provides a versatile approach for solving the critical radioresistance issue of hypoxic tumors.

## Abstract 2

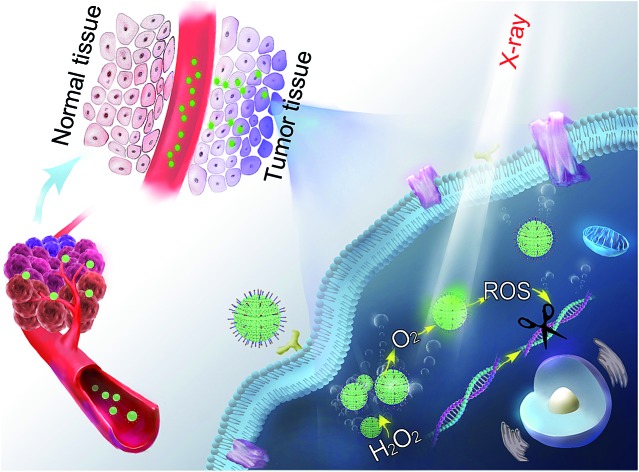
Tumor hypoxia typically occurs inside a solid tumor with an inadequate oxygen supply, sharply reducing the therapeutic efficiency of radiotherapy and significantly increasing the risk of local tumor recurrence.

## Introduction

Clinical findings indicate that 70% of cancer patients require radiotherapy (RT) at some stage of their disease course.^1^ RT utilizes high-intensity ionizing radiation (*e.g.*, X-rays, γ-rays) to induce DNA damage by generating reactive oxygen species (ROS), thus the therapeutic effect depends strongly on the oxygen (O_2_) level.^2^,^3^ However, the hypoxic nature of solid tumors seriously reduces the sensitivity of tumors to conventional RT.^4^ Moreover, only a small portion of the incident radiation energy of the radiation beam (*e.g.*, X-ray) can be absorbed by the tumor during RT, which further restricts the therapeutic outcomes and results in severe side effects. Thus, the development of effective methods to enhance RT and reduce side effects is of great significance.

H_2_O_2_ is an abundant metabolite in tumor tissues.^5^ Relatively high levels of H_2_O_2_ can maintain the malignant phenotype of tumors and further activate the expression of hypoxia-inducible factor-1 (HIF-1), which will aggravate hypoxia-induced tumor radioresistance.^6^,^7^ Catalytic decomposition of H_2_O_2_ should be an ideal strategy for enhanced cancer RT owing to its potency in elevating the O_2_ concentration in tumor tissues and relieving the tumor radioresistance. To date, few studies have reported the combination of O_2_ catalysts (*e.g.*, catalase or MnO_2_) and high-Z materials (radiosensitizers) to improve the effect of RT.^8^,^9^ The application of these compounds in cancer treatment remains limited by several factors, such as the poor stability, easy deactivation of natural enzymes, and the laborious process of assembly with an O_2_-catalyst and radiosensitizer. Therefore, a facile strategy for constructing an endogenous H_2_O_2_ biocatalytic platform that simultaneously integrates the O_2_ generation capability and radiosensitization effect is highly desirable.

Metal–organic framework nanoparticles (MOF NPs) are a group of porous materials self-assembled from metal ions or clusters and organic ligands *via* coordination interaction.^10^–^16^ Benefiting from their facile design, easy functionalization, and high biocompatibility,^17^–^20^ MOF NPs are considered ideal catalyst candidates: their unique open channel structure can offer abundant catalytic sites and facilitate the diffusion of diverse substrates, which can overcome the drawbacks of existing catalysts which have only limited active sites exposed on the surface. Furthermore, the diversity of clusters makes it feasible to integrate multiple functions into a single MOF NP. Accordingly, we speculate that MOF NPs should be promising materials that can simultaneously act as oxygen-catalysts and radiosensitizers.

**Scheme 1:**
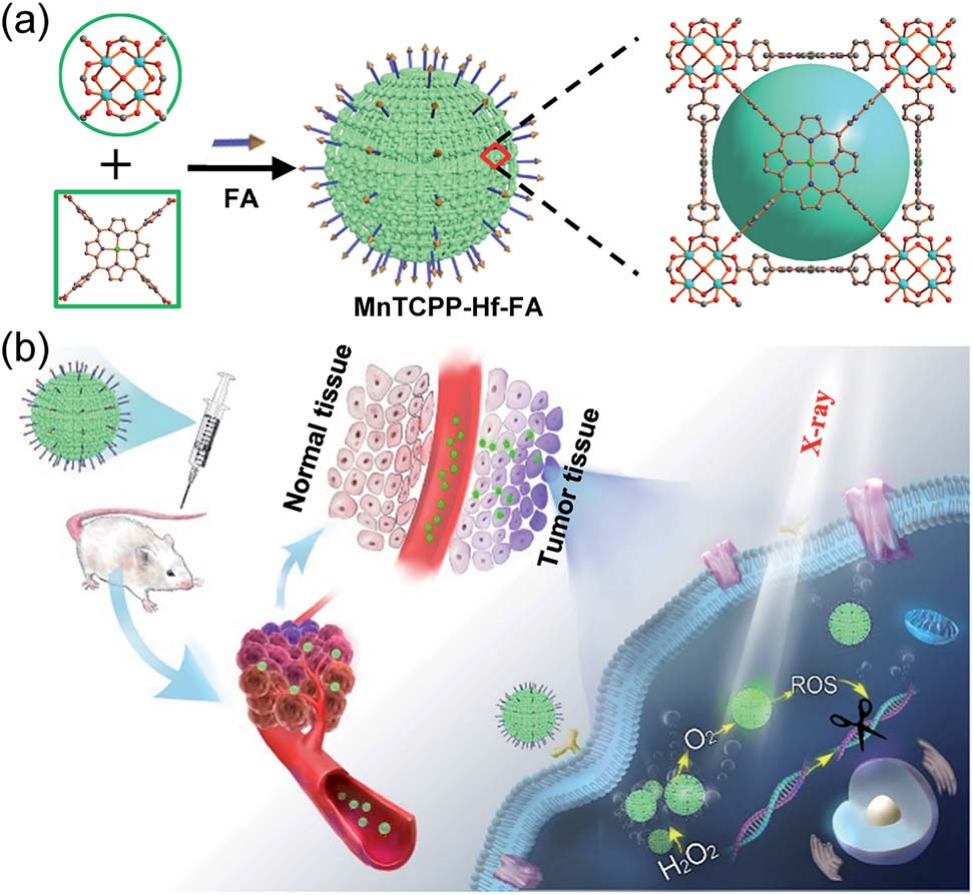
Schematic illustration of the process for synthesizing the nanosensitizer (a) and the representation of systemic and intracellular pathways and the action of the nanosensitizer (b).

In this study, we designed and synthesized a catalase-like MOF-based nanosensitizer (MnTCPP–Hf–FA MOF NPs) for tumor targeted RT. TCPP–Hf MOF NPs were facilely prepared from hafnium (Hf) clusters and tetrakis(4-carboxyphenyl)porphyrin (TCPP) ligands. Benefiting from the strong chelating interaction between manganese (Mn) ions and TCPP, the enzyme-like MnTCPP–Hf MOF NPs were obtained after chelating Mn ions. To endow the MOF NPs with tumor targeting capability, folic acid (FA), a cancer cell-targeting ligand,^21^–^23^ was further conjugated on the surface of the MOF NPs *via* coordination interaction. MnTCPP–Hf–FA exhibits a catalase-like property based on the reversible one-electron oxidation between Mn(iii)porphyrin and Mn(iv)porphyrin, which can effectively convert the endogenous H_2_O_2_ into O_2_. Due to the existence of the high-Z element Hf, MnTCPP–Hf–FA can further act as a radiosensitizer to enhance the radiotherapy–radiodynamic therapy. Particularly, the channel structure of MOF NPs facilitated the dispersion of catalytic sites and the rapid diffusion of O_2_ or ROS. Therefore, the prepared MOF-based radiosensitizer can realize effective tumor therapy with a single X-ray irradiation session, because it not only elevates the localized O_2_ concentration and relieves the hypoxia-dependent radioresistance but also has dose-enhanced capability during tumor RT (Scheme 1).

## Results and discussion

### Synthesis and characterization of the nanosensitizer

**Fig. 1:**
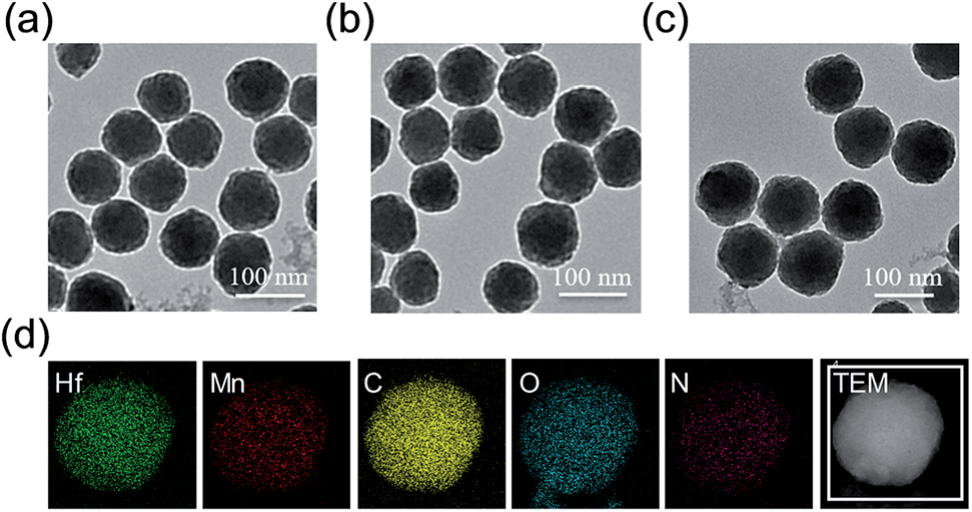
TEM images of TCPP–Hf (a), MnTCPP–Hf (b), and MnTCPP–Hf–FA (c). Element mapping of MnTCPP–Hf (d).

The *meso*-tetrakis(4-carboxylphenyl)porphyrin–hafnium MOF NPs (TCPP–Hf) were first constructed from the Hf-oxo cluster and TCPP ligand by a solvothermal method with further modifications.^24^ Next, manganese(iii) ions as the active centers were chelated into the porphyrin ring to form catalase-like MnTCPP–Hf MOF NPs. As shown in the transmission electron microscopy (TEM) images, TCPP–Hf displayed a spherical morphology (Fig. 1a). After Mn ion metalation, the morphology and size of MOF NPs were scarcely changed (Fig. 1b). Dynamic light scattering (DLS)^25^ showed that the average sizes were approximately 132 nm for TCPP–Hf and 138 nm for MnTCPP–Hf (Fig. S1a and b, ESI†). The crystal structure of MnTCPP–Hf obtained by powder X-ray diffraction (PXRD) was consistent with that of the as-reported sample of TCPP–Zr,^24^,^26^ demonstrating that the synthesized MnTCPP–Hf maintained a stable framework and adopted the MOF topology of Hf_6_(OH)_4_O_4_(MnTCPP–H_2_)_3_ (Fig. S2†). The Hf/Mn ratio in the MOF NPs was approximately 1.9 : 1 determined using the energy dispersive X-ray (EDX) spectrum (Fig. S3, ESI†) and inductively coupled plasma atomic emission spectroscopy (ICP-AES). The mass ratios of TCPP were determined to be 57.6 wt% by thermogravimetric analysis (TGA, Fig. S4, ESI†). At the same time, the elemental mapping images, UV-vis absorption spectra and Fourier transform infrared spectra (FTIR) further confirmed the successful synthesis at each step (Fig. 1d and S5 and S6, ESI†).

### Folic acid (FA) concentration optimization

The surface of MnTCPP–Hf was modified with FA by the coordination of the carboxylate end of the folate with the Hf clusters to obtain the catalase-like nanosensitizer (MnTCPP–Hf–FA).^27^,^28^ The content of FA on the surface of MOF NPs was firstly optimized. A series of NPs were prepared through adding different concentrations of FA (0, 1.25, 2.5, and 5.0 μM) in MnTCPP–Hf solution. Then, B16-F10 cells were incubated with MnTCPP–Hf–FA (10 μM) for 2 h, the intracellular Hf element was measured by ICP-AES. As shown in Fig. S7 in the ESI,† the Hf concentration in cells increased until the amount of FA reached 2.5 μM, so it was chosen in this study. The FA concentration was calculated to be 0.195 μM mg^–1^ MnTCPP–Hf (TCPP equiv.) using UV-vis spectra (Fig. S8, ESI†). Similar results were acquired by confocal laser scanning microscopy (CLSM, Fig. S9, ESI†). As shown in Fig. 1c and S1c,† the average diameter of MnTCPP–Hf–FA determined by DLS rose slightly to approximately 152 nm, further confirming the successful modification. In addition, the stability and biocompatibility of the MOF NPs were also studied *in vitro*. All results showed that the nanosensitizer remained relatively stable under physiological conditions and was well suitable for biomedical applications (Fig. S10–S12, ESI†).

### Evaluation of the enzyme-like activities of the nanosensitizer

**Fig. 2:**
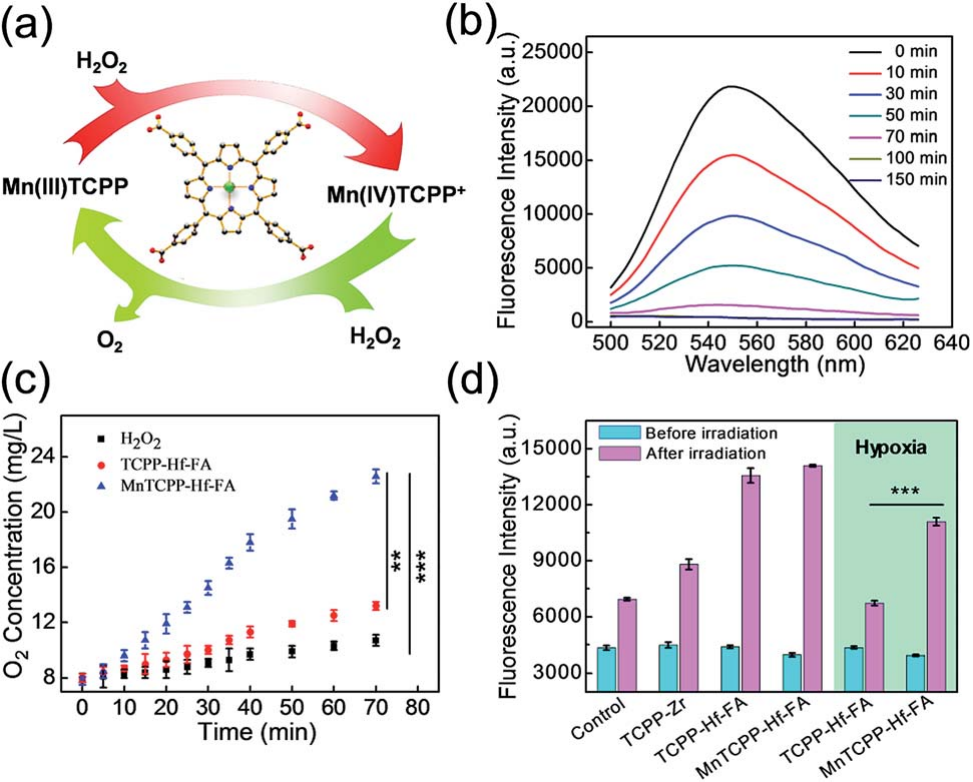
Schematic presentation of the catalase-like activities of the nanosensitizer (a). Fluorescence intensity of MI-H_2_O_2_ for H_2_O_2_ detection in the presence of MnTCPP–Hf–FA at different times (b). O_2_ generation in H_2_O_2_ solution at different times (c). Generation of ROS, as measured using the fluorescence intensity of SOSG with different nanoparticles under normoxic/hypoxic conditions (d) (Student's *t*-test, **p < 0.01, ***p < 0.001).

To evaluate the catalase-like activity of the nanosensitizer (Fig. 2a), the decomposition of H_2_O_2_ over time was investigated using a fluorescence probe, MI-H_2_O_2_.^29^ The fluorescence intensity gradually decreased during the initial 70 min in the presence of MnTCPP–Hf–FA compared with that of the control group (Fig. 2b and S13, ESI†), suggesting that most of the H_2_O_2_ was degraded. As expected, a significant elevation of O_2_ concentration over time under hypoxic conditions was also detected from the MnTCPP–Hf group using a portable dissolved oxygen meter (Fig. 2c). These results confirmed that the nanosensitizer possesses catalase-like activities and is able to decompose endogenous H_2_O_2_ to relieve hypoxia. Moreover, the O_2_ generation performance was also tested using other MOF NPs (Fig. S14†), which confirmed that the MnTCPP ligand was the appropriate choice. Additionally, the ROS generation efficiency of the nanosensitizer under X-ray irradiation was tested using the singlet oxygen sensor green (SOSG) agent.^30^ As shown in Fig. S15 in the ESI,† the fluorescence intensities increased with the dose in the solution containing MnTCPP–Hf–FA or TCPP–Hf–FA, suggesting that the as-synthesized MOF NPs could produce ROS under X-ray irradiation. No significant difference in ROS generation can be observed for MnTCPP–Hf–FA and TCPP–Hf–FA with the same dose of X-rays under normoxic conditions (Fig. 2d). However, under hypoxic conditions, MnTCPP–Hf–FA could produce approximately two-fold ROS compared with TCPP–Hf–FA, mostly attributed to the O_2_ generation capability of MnTCPP–Hf–FA.

### Evaluation of the enzyme-like activities of the nanosensitizer in cells

**Fig. 3:**
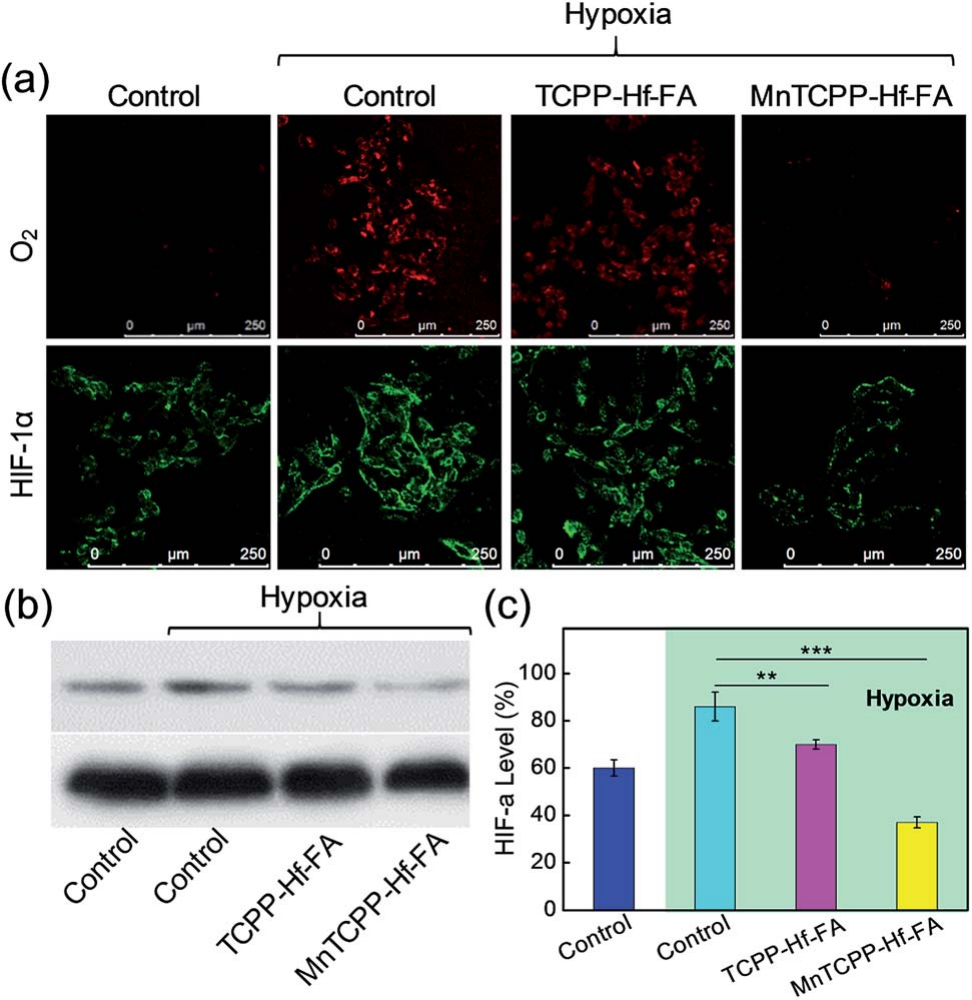
CLSM images of O_2_ generation and HIF-1α expression in B16-F10 cells after different treatments (a). Western blotting detection of HIF-1α expression in B16-F10 cells after different treatments. GAPDH was used as a loading control (b). Corresponding quantification of the gray value (c) (Student's *t*-test, **p < 0.01, ***p < 0.001).

To evaluate the intracellular catalase-like activity of MnTCPP–Hf–FA, the H_2_O_2_ levels in B16-F10 cells with different treatments were imaged by CLSM with Qcy7-H_2_O_2_ as an indicator.^31^ Bright green fluorescence was observed in the control group, indicating the high H_2_O_2_ level. However, the fluorescence intensity decreased dramatically in the MnTCPP–Hf–FA group compared with that in the TCPP–Hf–FA group, suggesting that MnTCPP–Hf–FA can cause intracellular H_2_O_2_ decomposition (Fig. S16, ESI†). [Ru(dpp)_3_]^2+^Cl_2_, which could be specifically quenched by O_2_, was employed to further verify O_2_ generation in living cells.^32^ As shown in Fig. 3a, clear red fluorescence was detected from the cells in the control or TCPP–Hf–FA groups under hypoxia, demonstrating the low oxygen levels in these cells. However, the fluorescence was quenched in the MnTCPP–Hf–FA group, indicating that MnTCPP–Hf–FA could catalyze H_2_O_2_ into O_2_. Correspondingly, the degree of hypoxia was investigated by monitoring HIF-1α protein levels using immunofluorescence staining and western blotting.^33^ Typically, the expression of HIF-1α, an oxygen-sensitive subunit, would be upregulated under hypoxic conditions. When the cells were pretreated with MnTCPP–Hf–FA, relatively low HIF-1α was detected, further suggesting that MnTCPP–Hf–FA could reduce hypoxia by O_2_ generation (Fig. 3a–c).

### Detection of ROS generation and DNA damage

**Fig. 4:**
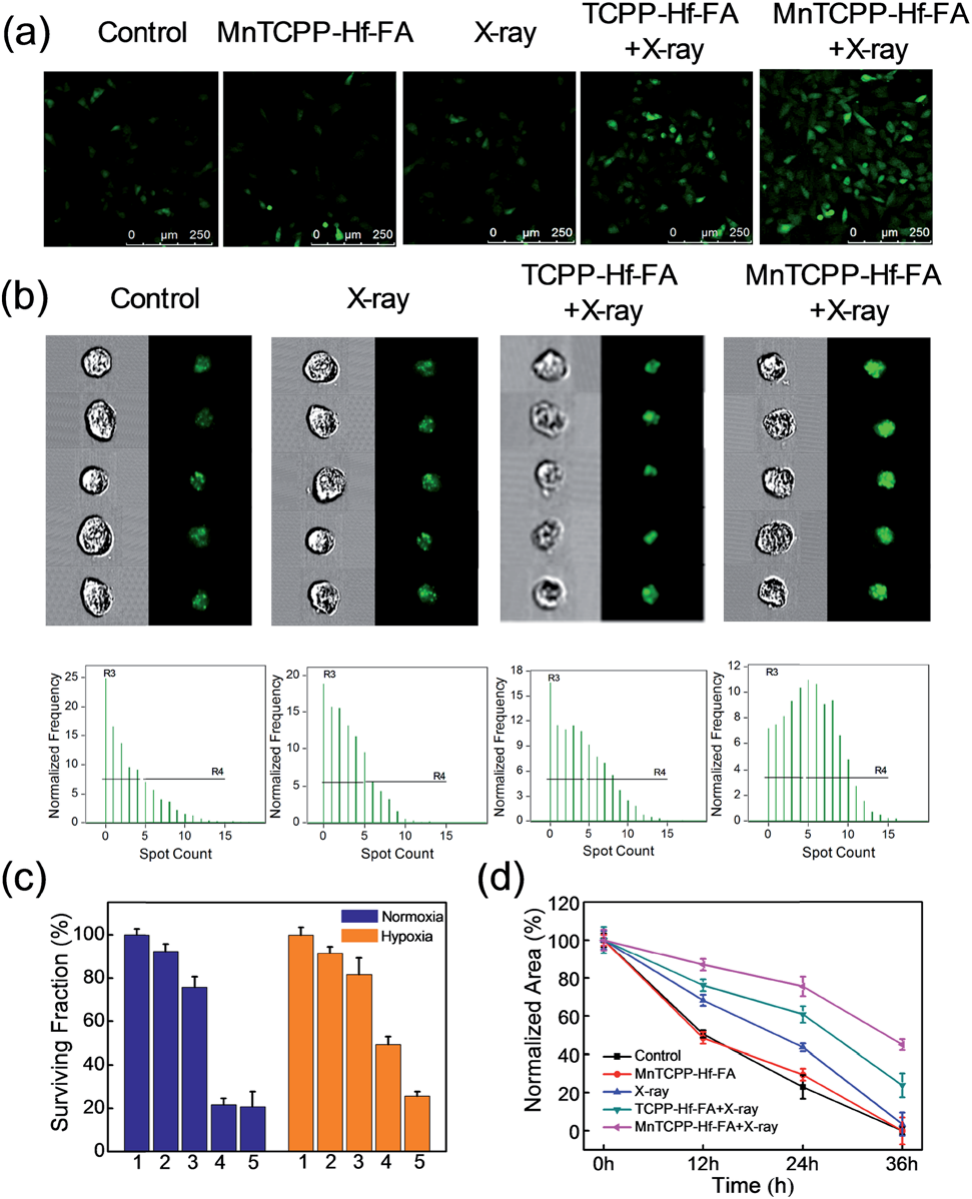
Generation of ROS measured by CLSM after different treatments of B16-F10 cells under hypoxic conditions after labeling with DCFH-DA (a). Verification of DNA double-strand breaks in B16-F10 cells *via* γ-H2AX immunofluorescence staining using imaging flow cytometry under hypoxic conditions (b). Corresponding quantitative data of the surviving cells subjected to different treatments and to the clonogenic assay (c). Normalized area of empty range with different treatments in a wound-healing assay of cells (d).

The ROS generation capacity of MnTCPP–Hf–FA in cells was also monitored using 2,7-dichlorofluorescein diacetate (DCFH-DA) as a probe.^34^ Under normoxic conditions, the fluorescence intensities were almost the same in the MnTCPP–Hf–FA-treated group and TCPP–Hf–FA-treated group upon 4 Gy of X-ray irradiation, demonstrating the same amount of ROS generation in these two groups (Fig. S17, ESI†). However, under hypoxic conditions, the fluorescence intensity of the MnTCPP–Hf–FA + X-ray-treated group was much higher than that of the TCPP–Hf–FA + X-ray-treated group, suggesting that the MnTCPP–Hf–FA-treated group could generate more ROS (Fig. 4a). These results indicated that MnTCPP–Hf–FA could generate adequate O_2_ to enhance RT. Next, the double-stranded DNA breaks were evaluated *via* immunofluorescence staining (γ-H2AX staining).^34^ DNA damage could be directly induced by ionizing radiation or indirectly induced by ROS.^35^ As shown in Fig. 4b and S18,† MnTCPP–Hf–FA + X-ray revealed markedly enhanced γ-H2AX immunofluorescence spots in B16-F10 cells under both normoxic and hypoxic conditions, indicating noticeable DNA damage after X-ray irradiation. These results demonstrated that MnTCPP–Hf–FA can cause a severe level of DNA double-strand breaks under hypoxic conditions, as expected.

### The effect on cell capability

To investigate whether the nanosensitizer could sensitize cancer cells to radiation under hypoxia, the clonogenic survival assay was performed in B16-F10 cells.^36^ As shown in Fig. 4c and S19,† the control and MnTCPP–Hf–FA groups had a similar and large number of cell colonies, demonstrating that the nanosensitizer has outstanding biocompatibility with a minimal effect. After 4 Gy of X-ray irradiation, TCPP–Hf–FA-treated cells showed a significant decrease in the colony number under normoxia but still had a relatively large number of colonies under hypoxia. Notably, MnTCPP–Hf–FA-treated groups displayed the least number of cell colonies under either normoxic or hypoxic conditions, revealed that MnTCPP–Hf–FA could effectively overcome hypoxia-associated radiation resistance. In addition, cell migration and invasion abilities were also evaluated by wound healing and transwell migration assays (Fig. 4d and S20–S22, ESI†). Under hypoxic conditions, the largest ratio of wound area (43.1%) and the lowest number of invaded cells were observed in the MnTCPP–Hf–FA + X-ray group, which further confirmed that MnTCPP–Hf–FA had greater capability to enhance tumor RT.

### *In vivo* therapeutic effect in xenograft tumor models

**Fig. 5:**
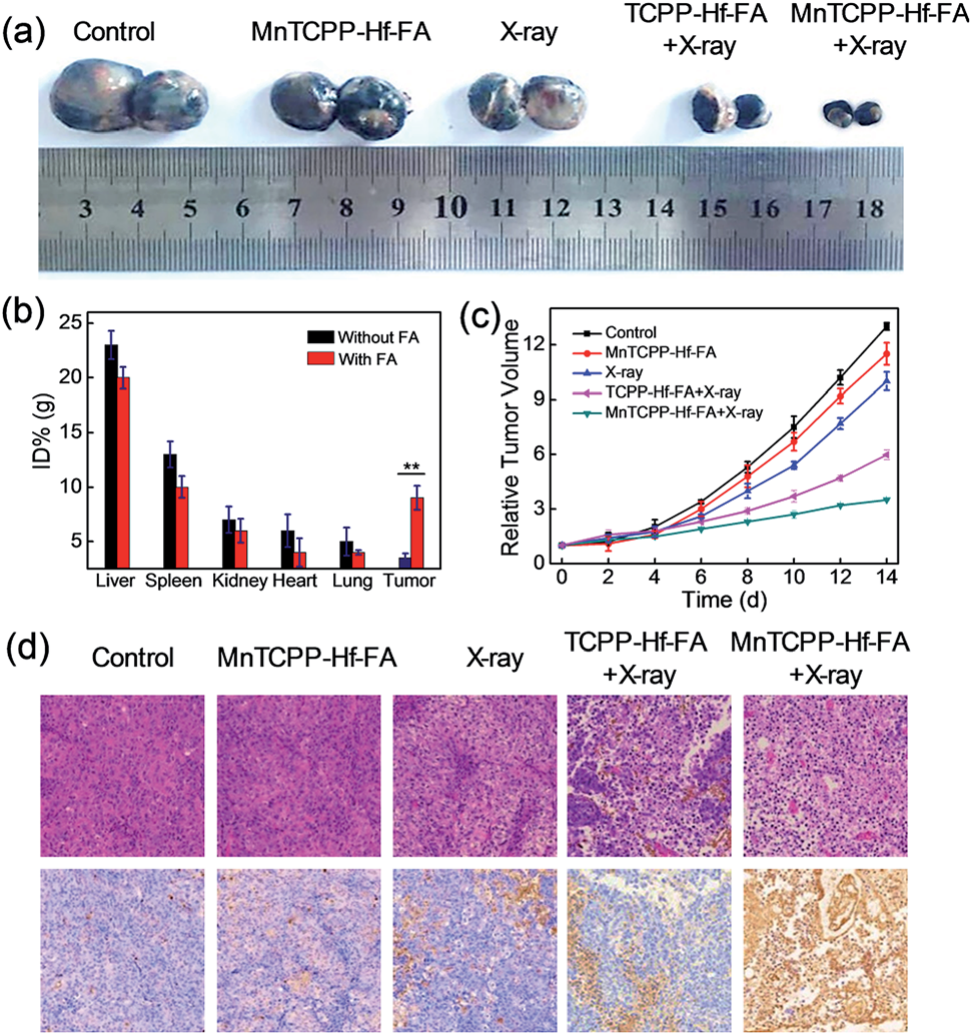
Photographs of the tumor after (14 days) the various indicated treatments (a). Biodistribution of MnTCPP–Hf with or without FA modification in different organs and tumors at 12 h posttreatment (Student's *t*-test, *p < 0.05, **p < 0.01) (b). Tumor volume changes of different treatment groups (c). H&E- and TUNEL-stained images of tumor slices obtained from different mice treated under various conditions for 12 h (d).

Encouraged by the superb *in vitro* performance, the efficiency of MnTCPP–Hf–FA against hypoxia tumors was further assessed in a melanoma tumor. Firstly, ICP-AES was employed to investigate the *in vivo* biodistribution of the nanosensitizer post injection into the mice (Fig. 5b and S23, ESI†). A significant portion, approximately 9.0% ID per g of injected Hf, was quantified in the tumor tissue in the MnTCPP–Hf–FA group, suggesting the targeting capability of MnTCPP–Hf–FA. Next, the *in vivo* antitumor ability of MnTCPP–Hf–FA was evaluated (Fig. 5a and c and S24, ESI†). No significant therapeutic effect was found in the MnTCPP–Hf–FA groups without X-ray irradiation compared with the control group. Only slight tumor inhibition was observed in the X-ray-treated groups due to the therapeutic effect of X-rays. Given the similar doses of X-ray irradiation, an obvious decrease in the tumor volumes was recorded for the MnTCPP–Hf–FA group compared with the TCPP–Hf–FA group, suggesting that MnTCPP–Hf–FA could provide enough oxygen in hypoxic tumors to enhance RT efficacy. Accordingly, a large area of apoptosis/necrosis of B16-F10 tumor cells was observed in the MnTCPP–Hf–FA + X-ray group using hematoxylin–eosin (H&E) staining and in terminal deoxynucleotidyl transferase dUTP nick end-labeling (TUNEL) images (Fig. 5d), further confirming the excellent therapeutic outcome of the nanosensitizer. During the treatment period, no obvious body weight changes or major organ damage in mice were observed after the various treatments (Fig. S25 and S26, ESI†). Moreover, no significant physiological difference in the routine blood parameters and serum biochemistry parameters was found in the mice treated with and without MOF NPs (Fig. S27, ESI†). These results illustrated that the nanosensitizer possessed minimum systemic toxicity. Furthermore, the *in vivo* metabolism results demonstrated that the nanosensitizer was gradually cleared in subsequent days (Fig. S28, ESI†). Importantly, a rather low concentration (approximately 2.7, 1.3, 0.8, 0.5, and 0.3% ID) of Hf was retained in the major mouse organs (heart, liver, spleen, lung, and kidney) after 7 days, indicating the intrinsic biocompatibility of nanoparticles (Fig. S29, ESI†).

### Tumor recurrence prevention

**Fig. 6:**
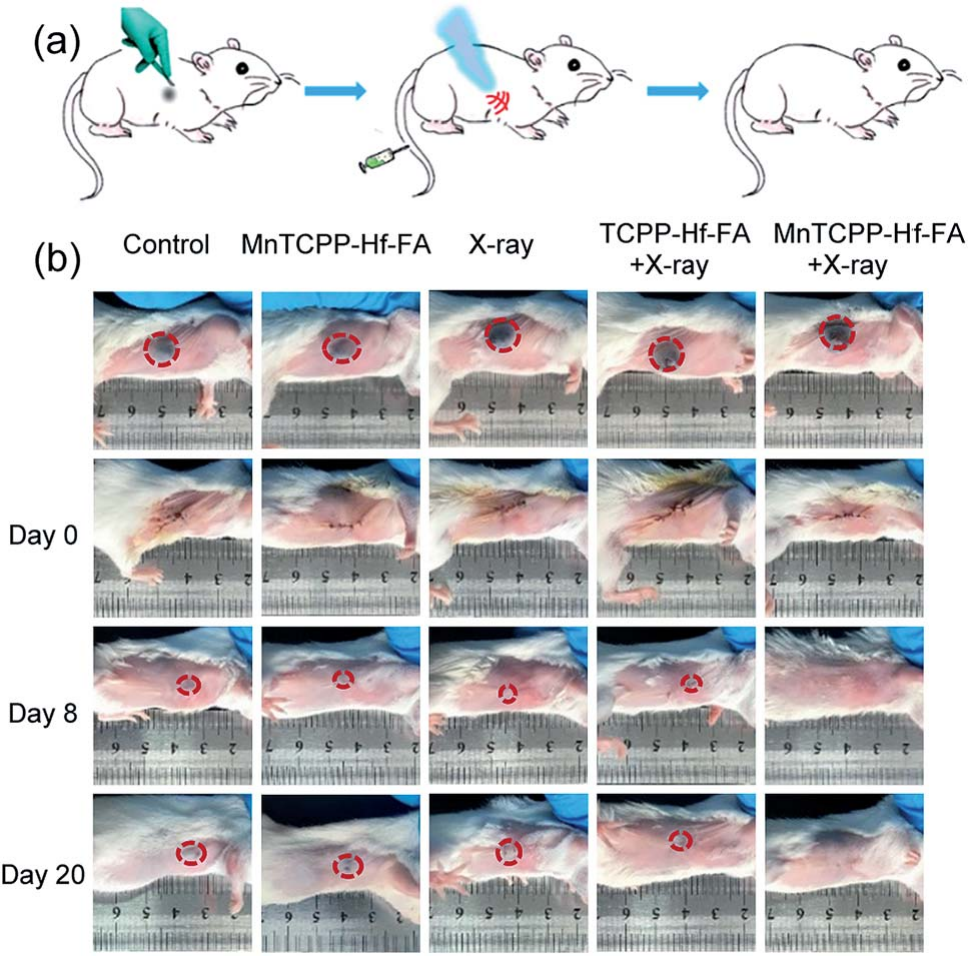
*In vivo* tumor recurrence after surgical resection. Schematic illustration of the *in vivo* tumor recurrence treatment process (a). Photographs of mouse tumor recurrence in different groups after tumor resection (b).

Postoperative recurrence of cancer frequently occurs during treatment, which usually increases the mortality rate.^37^,^38^ To evaluate the capability of the nanosensitizer in preventing postoperative recurrence, the model of tumor-burdened BALB/c mice following resection was used. As revealed in Fig. 6, certain degrees of recurrence were clearly observed in the first 8 days, and the tumor generally increased over time as measured up to 20 days in the control group or treated with MnTCPP–Hf–FA alone and X-ray irradiation alone. The TCPP–Hf–FA + X-ray group displays a small proportion of recurrence, while the MnTCPP–Hf–FA + X-ray group displayed no recurrence, indicating that the O_2_ generation was beneficial for radiosensitization and anti-recurrence. Moreover, no significant body weight changes were observed in all the groups up to 20 days (Fig. S30, ESI†), suggesting that different treatments have no significant effects on the normal growth of mice.

## Conclusions

In summary, to improve the effectiveness of RT for hypoxic tumors, an efficient therapeutic strategy was developed using MnTCPP–Hf–FA MOF NPs as a catalase-like nanosensitizer that integrates O_2_ generation *in situ* with enhanced radiosensitivity. The multifunctional MOF NPs could realize effective catalysis *in vivo*, and the framework structures ensure the highest density of active sites and feasible diffusion for O_2_ and ROS. Confocal images and western blotting indicated that the nanosensitizer could effectively decompose endogenous H_2_O_2_ into O_2_, successfully relieving the hypoxic conditions and inhibiting the activation of HIF-1α. The cell colony formation assay indicated that the cell survival rate was significantly lower than that in the nonenzymatic activity group under hypoxic conditions. The *in vivo* experiments showed that the catalase-like nanosensitizer could significantly inhibit tumor growth and prevent tumor recurrence with one application of X-ray irradiation. Compared to other MOF-based nanosensitizers, the designed nanoparticles for *in situ* O_2_-production and radiotherapy enhancement can be applied to solve the critical radioresistance issue of hypoxic tumors. We anticipate that the current strategy can provide more insights to design catalase-like nanosensitizers for biomedical applications.

## Ethical statement

All procedures of animal study were performed according to the Principles of Laboratory Animal Care (People's Republic of China) and the Guidelines of the Animal Investigation Committee, Biology Institute of Shandong Academy of Science, China. BALB/c mice (approximately 4–6 weeks old, ∼18 g) were housed under normal conditions with 12 h light and dark cycles and given access to food and water *ad libitum*.

## Conflicts of interest

The authors declare no competing financial interest.

## Supplementary Material

Supplementary information
