# Supplementary material for: Catalase-like metal–organic framework nanoparticles to enhance radiotherapy in hypoxic cancer and prevent cancer recurrence
Source: Chem Sci. 2019 Apr 25;10(22):5773–8. doi: 10.1039/c9sc00747d (PMC6563782; doi:10.1039/c9sc00747d)
Supplement: Supplementary file 1 [file SC-010-C9SC00747D-s001.pdf]

Electronic Supplementary Material (ESI) for Chemical Science.
This journal is © The Royal Society of Chemistry 2019

Electronic Supplementary Information (ESI)

Catalase-like metal-organic framework nanoparticles to enhance radiotherapy in hypoxic cancer and prevent cancer recurrence

Yuanyuan Chen,[a] *Hui Zhong,*[a] *Jianbo Wang,*[b] *Xiuyan Wan,*[a] *Yanhua Li,*[a] *Wei Pan,*[a] *Na Li,*[*a] *and Bo Tang*[*a]

[a]College of Chemistry, Chemical Engineering and Materials Science, Collaborative Innovation Center of Functionalized Probes for Chemical Imaging in Universities of Shandong, Key Laboratory of Molecular and Nano Probes, Ministry of Education, Institute of Molecular and Nano Science, Shandong Normal University, Jinan 250014, P. R. China.

[b]Radiation Department, Qilu Hospital of Shandong University, Jinan 250100, P. R. China.

E-mail: lina@sdnu.edu.cn; tangb@sdnu.edu.cn

Experimental Procedure

Materials. Hafnium (IV) chloride ($HfCl_4$) was purchased from Sigma-aldrich; 4,4,4,4-(Porphine-5,10,15,20-tetraly) tetrakis (benzoic acid) (TCPP), Singlet oxygen sensor green (SOSG) agent, Tris (4, 7-diphenyl-1,10-phenanthroline) ruthenium (II) dichlo-ride ($[Ru(dpp)_3]^{2+}Cl_2$) was obtained from Macklin (Shanghai, China); 2′, 7′-dichlorofluorescein diacetate (DCFH-DA) was obtained from Beyotime (Nantong, China). 3-(4,5-dimethyl-thiazol-2-yl)-2,5-diphenyltetrazolium bromide (MTT) was purchased from Sigma Chemical Company. The murine skin melanoma cells (B16-F10) were obtained from Aoluo Biotechnology Co., Ltd KeyGEN biotechnology (Shanghai, China). Glass bottom dishes were purchased from Cellvis (Mountain View, CA). All other reagents and solvents were analytical grade. Sartorius ultrapure water (18.2 $M\Omega \cdot cm^{-1}$) was used for experiments.

Instruments. The transmission electron microscopy (TEM) was carried out on a JEM-2100 electron microscope. Fluorescence spectra were obtained with FLS-980 Edinburgh. All pH measurements were performed with a pH-3c digital pH-meter (Shanghai LeiCi, China) with a combined glass-calomel electrode. Confocal fluorescence imaging studies were performed with a TCS SP5 confocal laser scanning microscopy (Leica Germany) with an objective lens (×20). The crystal structure of the samples was determined by powder X-ray diffraction (PXRD) patterns (Bruker D8, Germany). The changes of structure were characterized by Fourier transformed infrared (FTIR) spectrophotometer (Therm Fisher Scientific, America); The Hf^{4+} content was detected by ICP-AES (Thermo Scientific iCAP 7400); Radiotherapy was carried out on a medical linear accelerator (Varian 23 EX, America) at a power of 6 MV with dose rate of 400 cGy/min and the source skin distance = 100 cm.

Synthesis of MnTCPP-Hf. TCPP-Hf was synthesized according to a previous report with some modifications. First, 4 mg of $HfCl_4$ and 6 mg of TCPP were dispersed in 5 mL of DMF and were stirred overnight in the dark. Second, 400 μL of acetic acid was added to the mixture, followed by

incubation in an oven at 80 °C for 2 h. Thereafter, 6 mL of free DMF was mixed with the solution mentioned above for another 24 h. Finally, after being cooled to room temperature, the final product of TCPP-Hf was obtained by centrifugation, followed by washing with DMF, triethylamine/ethanol (v/v = 1/20) and ethanol three times each. For the synthesis of MnTCPP-Hf, TCPP-Hf (11.85 mg) and $MnCl_2$ (20.15 mg) in DMF were refluxed for one hour under an argon atmosphere. After being cooled to room temperature, the dark-green solid product was collected by centrifugation and washed with DMF, ethanol and water three times each.

Folic acid (FA) modification of MnTCPP-Hf. The dried MnTCPP-Hf (1.24 mg) was resuspended in 1 mL of distilled water to obtain the final concentration of 1 mM (The molecular weight of MnTCPP-Hf based on each porphyrin is 1,240.91 g/mol). Next, different amounts of FA (0 μmol, 0.125 μmol, 0.25 μmol, 0.5 μmol) were first dissolved in 50 μL of DMSO and then added to 1 mL of MnTCPP-Hf solution. After vigorous stirring for 30 min, the excessive free folic acid was removed by centrifugation and washed with DMSO/water (v/v = 1/99). To determine the content of FA modified on the surface of MOF NPs, the supernatant after the first centrifugation was collected and diluted to 1/10. At the same time, the original concentrations of FA were deemed to be 0 μM, 1.25 μM, 2.5 μM, 5.0 μM. The UV-vis absorption spectrum of the characteristic peak at 280 nm was measured, and the content of FA was calculated using subtraction *via* the standard linear calibration curve of FA.

Degradation experiment in vitro. *In vitro* degradation profiles of the MnTCPP-Hf-FA MOF NPs were assessed by detecting the accumulated degradation content of Hf by ICP-AES. Typically, the MOF NPs were added into different simulated physiological solution (saline, cell lysis solution), respectively. The testing solution was put into a water bath under magnetic stirring slowly. At given time, a small amount of degradation solution was taken out for ICP-AES test. The leaching test of

Mn in saline or HEPES buffer solution: the MnTCPP-Hf-FA MOF NPs were added into a dialysis bag with cut-off molecular weight (MV) of 500 Da and dialyzed against saline or HEPES buffer solution under magnetic stirring. At given time, a small amount of solution was taken out for ICP-AES test.

Cell viability assay. B16-F10 cells were cultured in 96-well microtiter plates and were incubated at 37 °C for 24 h. Next, B16-F10 cells were incubated with the nanoparticles (Control, TCPP-Hf-FA, MnTCPP-Hf-FA) at different concentrations (0, 20, 40, 60 and 80 μM, TCPP equiv) for 24 h. Thereafter, 150 μL MTT solution (0.5 mg/mL) was added to each well. After 4 h of treatment, the MTT solution was discarded, and 150 μL of DMSO was added to dissolve crystals. Finally, the absorbance was measured at 490 nm using an RT 6000 microplate reader.

Hemolysis assay. Fresh RBCs (red blood cells) were collected from BALB/c mice and washed with saline for three times. The RBCs suspension (0.5 mL) was added to 0.5 mL saline containing different concentrations of MnTCPP-Hf MOF NPs to obtain samples with the final concentration of 10, 20, 40, 60, 80 μM. The RBCs suspension (0.5 mL) added with saline (0.5 mL) or water (0.5 mL) were used as the negative control and positive control, respectively. The suspension was mixed gently, left at room temperature for 12 h, and then centrifuged. The supernatant (100 μL) was transferred to a 96-well plate and the absorbance value of hemoglobin at 570 nm was measured. The percentage of hemolysis was calculated as follows:

Hemolysis % = [(sample absorbance – background absorbance) / (positive control – negative control)] × 100%.

Detection of H_2O_2 decomposition and O_2 generation. To determine the capability of the catalytic decomposition of H_2O_2, 2.5 mM of H_2O_2 and 500 μM of MnTCPP-Hf-FA were mixed in HEPES (20 mM pH 7.4) at 37 °C. At different times (0, 10, 30, 50, 70, 100, 150 min), 100 μL of the mixture

was collected, centrifuged and added to the working solution (980 μL of PBS, 114 μL of DMSO and 6 μL of MI-H_2O_2). The fluorescence spectra were recorded at $\lambda_{ex}/\lambda_{em}$ = 400/550 nm. Measurement of O_2 production: 50 μmol different nanoparticles (MnTCPP-Hf-FA NMOF, MOF-525, MOF-525-Zn, MOF-525-Co, MOF-525-Fe) was incubated with 150 μmol of H_2O_2 at 37 °C in 50 mL free-oxygen HEPES buffer, followed by measuring the O_2 concentration with a dissolved oxygen meter at different times.

Detection of ROS. 1) First, 10 μL of SOSG (5 mM) was added to the mixture (control, TCPP-Hf-FA (20 μM, TCPP equiv) and MnTCPP-Hf-FA (20 μM, TCPP equiv)). Second, five different doses (0 Gy, 2 Gy, 4 Gy, 6 Gy, 8 Gy or 10 Gy) were given. Finally, the fluorescence intensity was recorded after centrifugation of the mixture. 2) Ten microliters of SOSG (4 mM) was added to the mixture (control, Zr-TCPP (20 μM, TCPP equiv), TCPP-Hf-FA (20 μM, TCPP equiv) and MnTCPP-Hf-FA (20 μM, TCPP equiv)). The fluorescence intensity was measured after 8 Gy of X-ray irradiation. Moreover, the ability of ROS generation by TCPP-Hf-FA and MnTCPP-Hf-FA was also evaluated under free-oxygen condition.

Optimization of FA. B16-F10 cells were plated on 35-mm glass-bottomed dishes for 24 h. A series of MnTCPP-Hf-FA (20 μM, TCPP equiv) containing various FA was then incubated with the above cells in culture medium in 5% CO_2 at 37 °C for 2 h. Next, the cells were washed with PBS three times before trypsinization. Thereafter, the cell pellet was collected by centrifugation and solubilized in digesting solutions ($V_{HClO_4}/V_{HCl}/V_{HNO_3}$ = 2/1/3) overnight. For inductively coupled plasma atomic emission spectroscopy (ICP-AES) analysis, the above solution was diluted by Milli-Q water to 10 mL, and the concentration of Hf^{4+} was detected in each sample. Additionally, the cellular uptake was also tested by confocal laser scanning microscopy (CLSM). After cells were incubated with different nanoparticles (MnTCPP-Hf (20 μM, TCPP equiv) and MnTCPP-Hf-FA (20 μM, TCPP equiv)

containing FA (2.5 μmol) for 2 h, confocal images were captured with 405 nm excitation and collected with the range of 600 nm to 700 nm.

Detection of H_2O_2 and O_2 in cells. The cells were first incubated with different nanoparticles (TCPP-Hf-FA (20 μM, TCPP equiv) and MnTCPP-Hf-FA (20 μM, TCPP equiv)) for 12 h in 5% CO_2 at 37 °C, followed by treatment with 150 μM H_2O_2 for 1 h. Thereafter, the cells were washed with PBS three times and further incubated for another 4 h. Finally, the laser confocal images were captured by CLSM under Qcy7-H_2O_2 labeled with cells (λ_{ex} = 488 nm and λ_{em} = 600 - 700 nm). Moreover, the detection of intracellular O_2 was also carried out. The cells were first incubated with different nanoparticles for 6 h at 37 °C in 5% CO_2 and then were washed with PBS three times. Certain cells were subjected to anaerobic condition in an incubator (MCO-15AC, Sanyo, Tokyo, Japan) for another 6 h. Before the laser confocal images were captured by CLSM with the excitation of 488 nm, the cells were stained with tris(4,7-diphenyl-1,10-phenanthroline) ruthenium (II) dichloride ($[Ru(dpp)_3]^{2+}Cl_2$) for 6 h.

HIF-1α immunostaining and western blotting. The cells were first incubated with different nanoparticles (TCPP-Hf-FA (20 μM, TCPP equiv) and MnTCPP-Hf-F (20 μM, TCPP equiv)) for 12 h at 37 °C in 5% CO_2, and then, certain cells were subjected to anaerobic condition for 6 h. After fixation with 4% paraformaldehyde for 20 min, the cells were stained with antibodies against HIF-1α (ab190197; Abcam, Cambridge, MA, USA). The cell fluorescence was analyzed by CLSM with the excitation of 488 nm. Moreover, the content of HIF-1α was also quantified by western blotting. The cell samples were prepared according to the mentioned methods above and were monitored by enhanced chemiluminescence using the Gel Doc system.

Detection of ROS in cells. The cells were first incubated with different nanoparticles (TCPP-Hf-FA (20 μM, TCPP equiv) and MnTCPP-Hf-FA (20 μM, TCPP equiv)) for 12 h in 5% CO_2 at 37 °C, and

then, certain cells were subjected to anaerobic condition for 6 h. After labeling with DCFH-DA for 20 min, the cells were subjected to X-ray irradiation of 4 Gy. Finally, the confocal images were immediately obtained by CLSM with 488-nm excitation. In addition, the kinds of ROS generation by MnTCPP-Hf-FA under X-ray irradiation were also clarified in vitro/in vivo. Methylene blue (MB) and coumarin were chosen as fluorescence indicator to selectively trap •OH in chemical and intracellular systems, respectively. Moreover, the $^1O_2$ generation of MnTCPP-Hf-FA upon X-ray irradiation were determined by 1,3-Diphenylisobenzofuran (DPBF) and singlet oxygen sensor green (SOSG) in chemical and intracellular systems, respectively.

DNA damage evaluation. DNA damage was detected using the γ-H2AX Phosphorylation Assay Kit. Briefly, the cells were first incubated with different nanoparticles (TCPP-Hf-FA (20 μM, TCPP equiv) and MnTCPP-Hf-FA (20 μM, TCPP equiv)) for 6 h in 5% CO_2 at 37 °C, and then, certain cells were subjected to anaerobic condition for 12 h. After irradiation with 4 Gy of X-rays, the cells were further incubated for 4 h. Next, the cells were fixed with 4% paraformaldehyde for 20 min and were eventually stained with FITC-conjugated anti-phospho-histone γ-H2AX (Ser139). Finally, an imaging flow cytometer (Amnis Corporation) was applied to record the cell images with an excitation of 488 nm for FITC. IDEAS® image analysis software (Amnis) was used to analyze the images.

Cell migration and invasion assay. (1) The cells were seeded into 60-mm dishes and were incubated at 37 °C under 5% CO_2 in RPMI 1640 for 12 h. The cells were further subjected to different conditions, and the dose of X-rays was 4 Gy. The monolayer was wounded using a 10-μL sterile pipette tip, and cell images were taken at time 0, 12, 24 and 36 h post-wounding. The area of wound healing was calculated using AJ-VERT software, and each experiment was performed in triplicate. The hypoxic conditions were applied in an incubator for 6 h. (2) Cell invasion assays were

conducted using Matrigel-coated invasion chambers with an 8-μm pore size in 24-well plates (BD Biosciences). B16-F10 cells were treated as mentioned method above; 2×10^4 cells were added into the upper compartment and further incubated for 24 h. After removing the noninvasive cells on the upper surface of the membrane, the invasive cells were fixed with 4% paraformaldehyde and were stained with 0.2% crystal violet before counting the number of invaded cells under a microscope. Hypoxic conditions were applied in an incubator for 6 h.

Clonogenic assay. First, 8×10^3 cells were seeded in 35-mm dishes at 37 °C under 5% CO_2 for 12 h, and then some of them were placed in an incubator for 6 h. After incubation with various nanoparticles (20 μM, TCPP equiv) for another 12 h, the cells were subjected to 4 Gy X-ray irradiation. Next, the cells were incubated at 37 °C under 5% CO_2 for 10 days before being fixed with 4 % polysorbate and stained with crystal violet. Cell colonies were counted only if they contained more than 50 cells. The surviving fraction = (surviving colonies) / (cells seeded × plating efficiency). The mean surviving fraction was obtained from three replicates.

Tumor model establishment.

The B16-F10 melanoma model was used as an example to evaluate the therapeutic effect. To this end, 1×10^7 B16-F10 cells in 100 μL of serum-free RPMI 1640 medium were injected subcutaneously into the right axillary region of BALB/c mice. After the tumor size had reached approximately 80-100 mm^3, the mice were used in subsequent experiments.

***In vivo* therapeutic experiments in xenograft tumor models.** First, the mice were divided into five different groups: (i) Control, (ii) MnTCPP-Hf-FA, (iii) X-ray, (iv) TCPP-Hf-FA+X-ray, and (v) MnTCPP-Hf-FA+X-ray. The MOF NPs were diluted in physiological saline to 5 mg/mL, and 50 μL was administered to each mouse by intravenous injection. Next, 4 Gy of X-ray was irradiated on the tumor site after injection for 12 h. The tumor sizes and body weights were measured every other day

for 14 days after treatment (tumor volume = $W^2 \times L/2$, W = width, L = length). The relative tumor volumes were calculated for each mouse as V/V_0 (V_0 was the tumor volume when the treatment was initiated. (2) H&E staining and TUNEL staining of tumor slides: 12 h after different treatments, the mice were sacrificed, and the tumors were harvested to use for hematoxylin and eosin (H&E) staining and TUNEL staining; H&E staining of five major organs (liver, lung, spleen, kidney, and heart): 7 days after different treatments, the mice were sacrificed, and five major organs (liver, lung, spleen, kidney, and heart) were harvested to use for H&E staining.

***In vivo* hemocompatibility**. In vivo hemocompatibility of MnTCPP-Hf-FA was evaluated using serum biochemistry and routine blood test using BALB/c mice. The MOF NPs were diluted in physiological saline to 5 mg/mL, and 50 μL was administered to each mouse by intravenous injection. After 0.5, 1 and 7days feeding, mice were anesthetized to collect blood. Blood supernatant harvested via centrifugation was used to analyze the serum biochemistry parameters. Two hepatic function indicators (ALT, AST), two kidney function indicators (BUN, CR) were measured. Heparin stabilized blood was used for routine blood test. Parameters including white blood cell (WBC), red blood cells (RBC), platelet thrombocytocrit (PCT), mean corpuscular hemoglobin (MCH), red cell distribution width (RDW), hematocrit (HCT), mean corpuscular volume (MCV), hemoglobin (HGB), mean corpuscular hemoglobin concentration (MCHC), and platelet (PLT). These analyses were provided by Wuhan servicebio technology CO., LTD.

Tumor recurrence prevention. B16-F10 melanoma cells were first injected subcutaneously into the right axillary region. When the tumor volumes reached ~300 mm^3 in approximately 10 days, the tumors were removed through surgery, leaving residual microtumors. Next, the mice were divided into five different groups: (i) Control, (ii) MnTCPP-Hf-FA, (iii) X-ray, (iv) TCPP-Hf-FA+X-ray, and (v) MnTCPP-Hf-FA+X-ray. The MOF NPs were diluted in physiological saline to 5 mg/mL,

and 50 μL was administered to each mouse by intravenous injection. Then, 12 h postinjection, the tumor site was irradiated with 4 Gy of X-rays. The body weights were measured every other day after treatment.

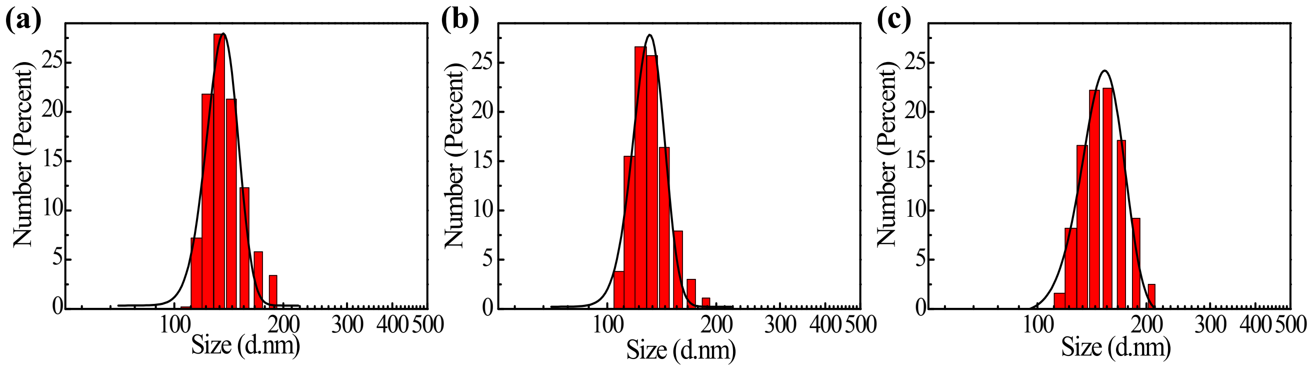

Fig. S1. The size distribution through DLS analysis in HEPES buffer (20 mM, pH 7.4) of TCPP-Hf (a); MnTCPP-Hf (b) and MnTCPP-Hf-FA (c).

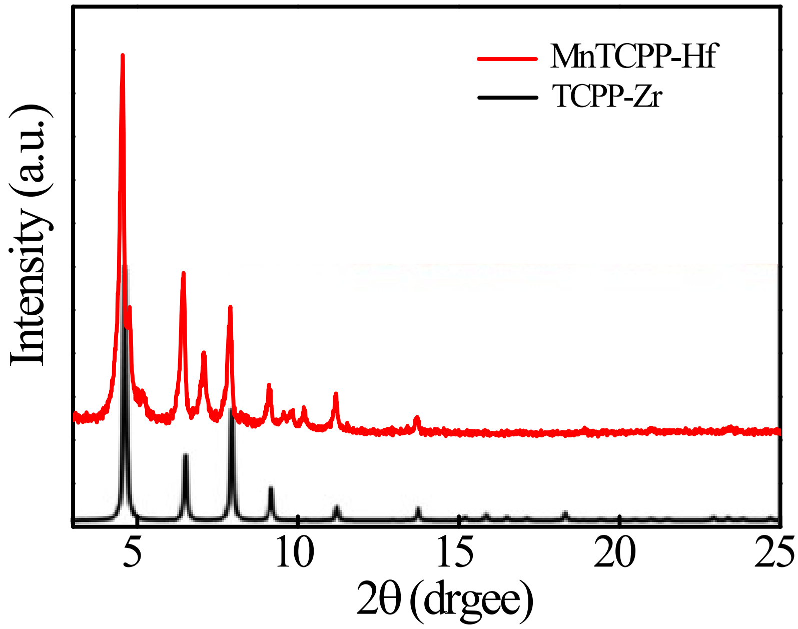

Fig. S2. The powder X-ray diffraction (PXRD) patterns of MnTCPP-Hf MOF NPs and as-reported TCPP-Zr MOF NPs.

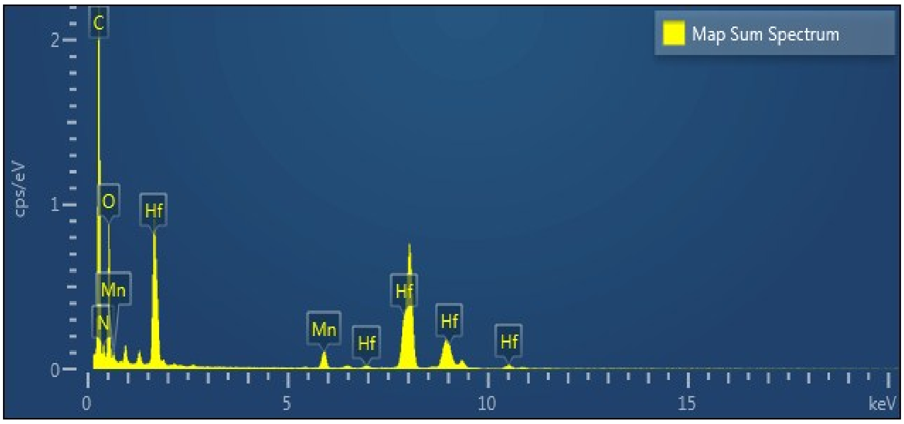

Element	Line Type	k factor	Absorption Correction	Wt%	Wt% Sigma
C	K series	2.50675	1.00	56.23	0.41
N	K series	3.14061	1.00	1.89	0.30
O	K series	1.86867	1.00	16.71	0.26
Mn	K series	1.17370	1.00	3.17	0.10
Hf	M series	1.57515	1.00	22.00	0.34
Total:				100.00	

Fig. S3. The EDX spectrum of MnTCPP-Hf.

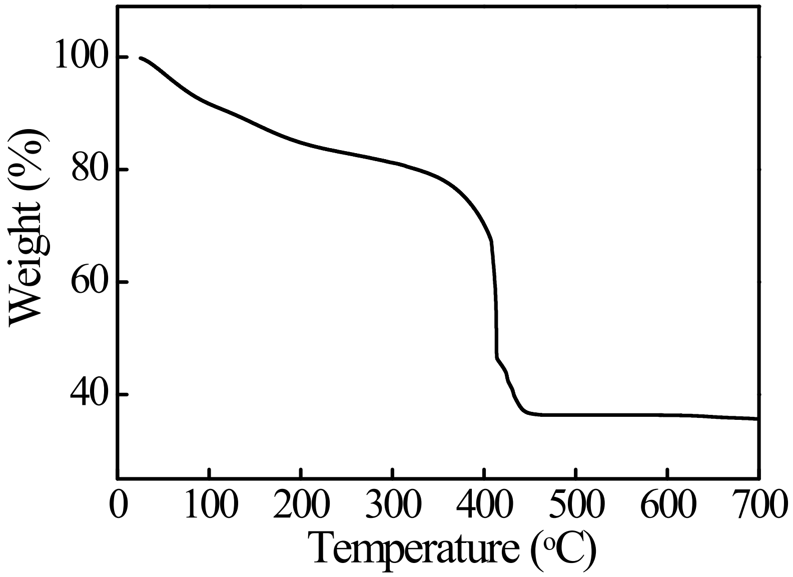

Fig. S4. Thermo-gravimetric analysis of MnTCPP-Hf MOF NPs.

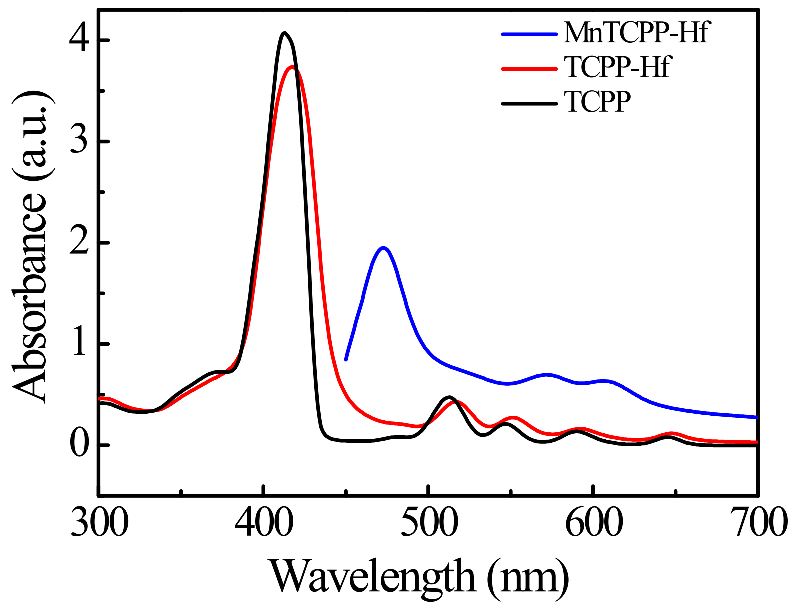

Fig. S5. UV-vis absorbance spectra of TCPP, TCPP-Hf and MnTCPP-Hf. The number of Q-bands of MnTCPP-Hf decreased from four to two in the range of 500-700 nm, which attributed to the binding of the Mn ions by the nitrogen atoms within porphyrin.

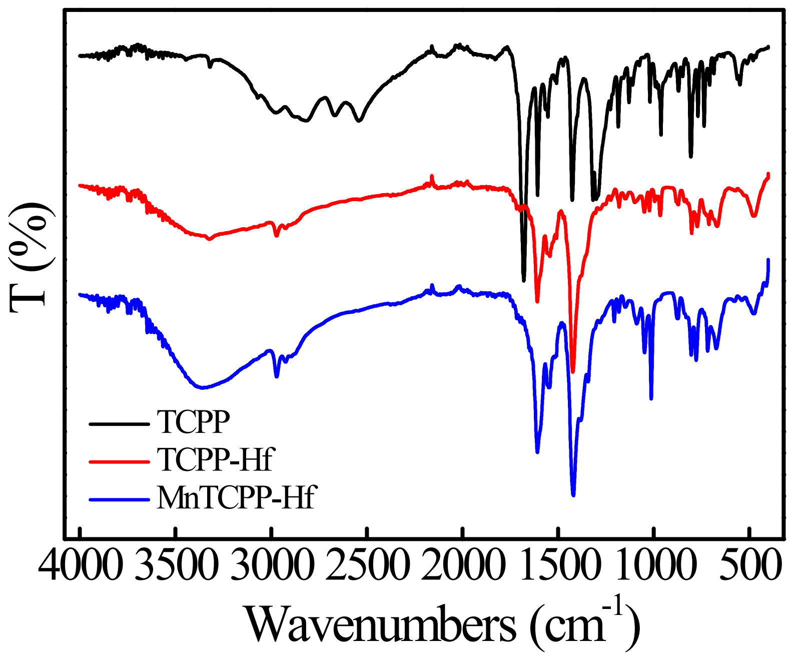

Fig. S6. FTIR spectra of TCPP, TCPP-Hf and MnTCPP-Hf.

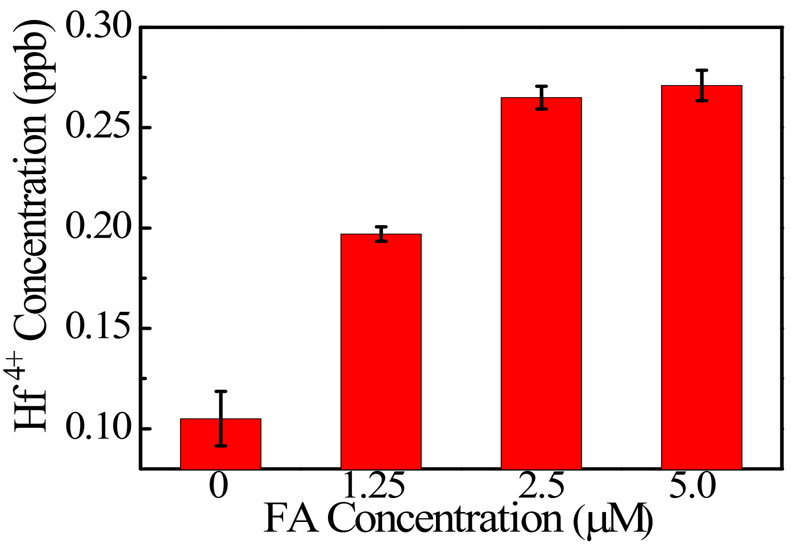

Fig. S7. Cellular uptake of MnTCPP-Hf-FA with different FA concentrations and the incubation time of 2 h; Data are based on ICP-AES analysis of the Hf concentration internalized in the B16-F10 cells.

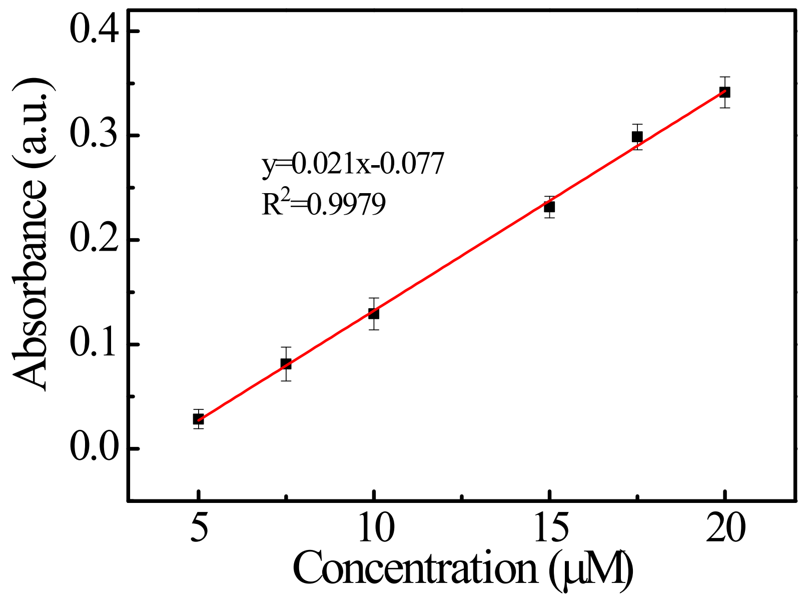

Fig. S8. Standard linear calibration curve of FA.

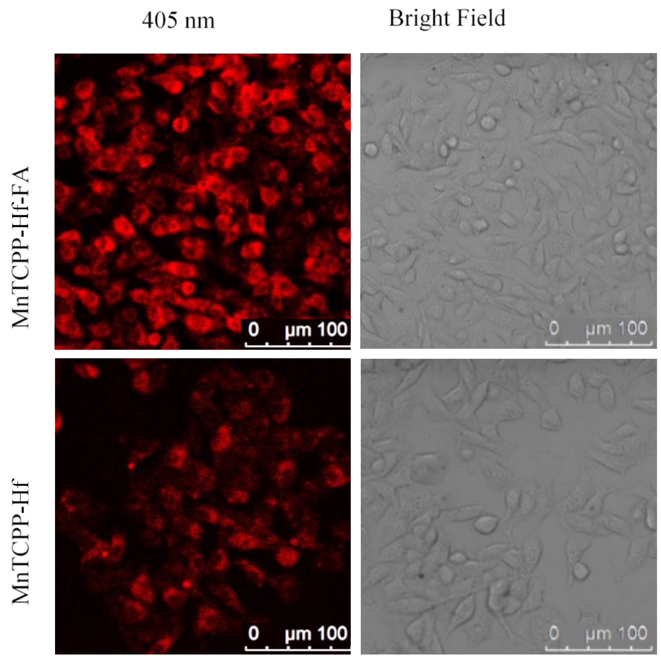

Fig. S9. Confocal images of B16-F10 cells incubated with MnTCPP-Hf or MnTCPP-Hf-FA (with or without folic acid modification) for 2 h by CLSM.

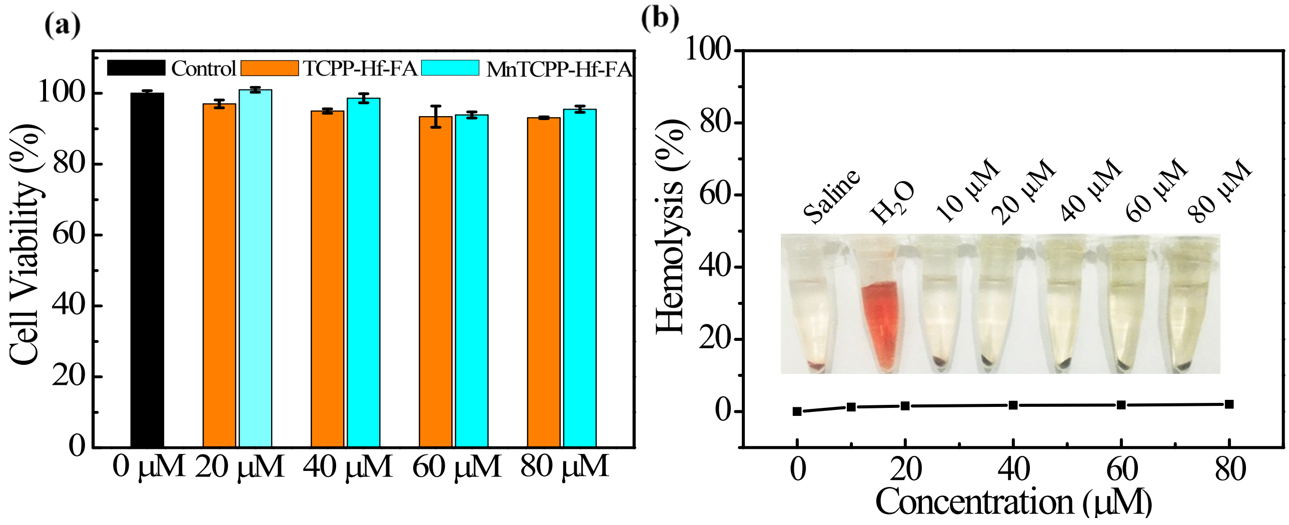

Fig. S10. (a) In vitro cell viability test by MTT assay after B16-F10 cells were treated differently for 24 h. (b) Blood hemolysis using MnTCPP-Hf-FA MOF at concentrations from 10 to 80 μM. Water was used as a positive control and saline was used as a negative control.

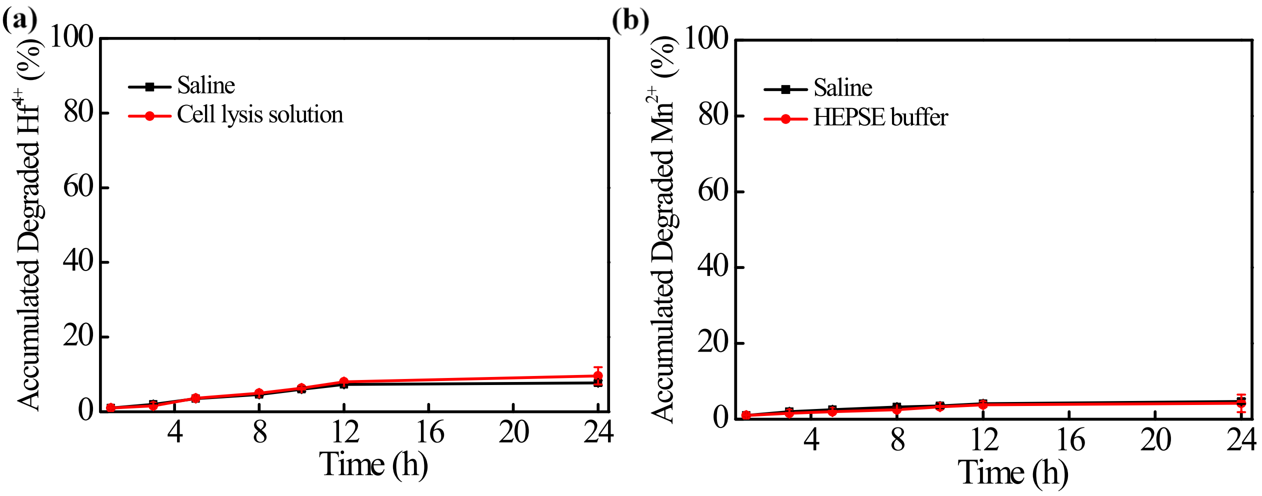

Fig. S11. (a) Accumulated degradation of Hf^{4+} components in MnTCPP-Hf-FA MOF at different simulated physiological solution (saline, cell lysis solution) monitored by ICP-AES. (b) Accumulated degradation of Mn^{2+} components in MnTCPP-Hf-FA MOF in saline or HEPES buffer monitored by ICP-AES.

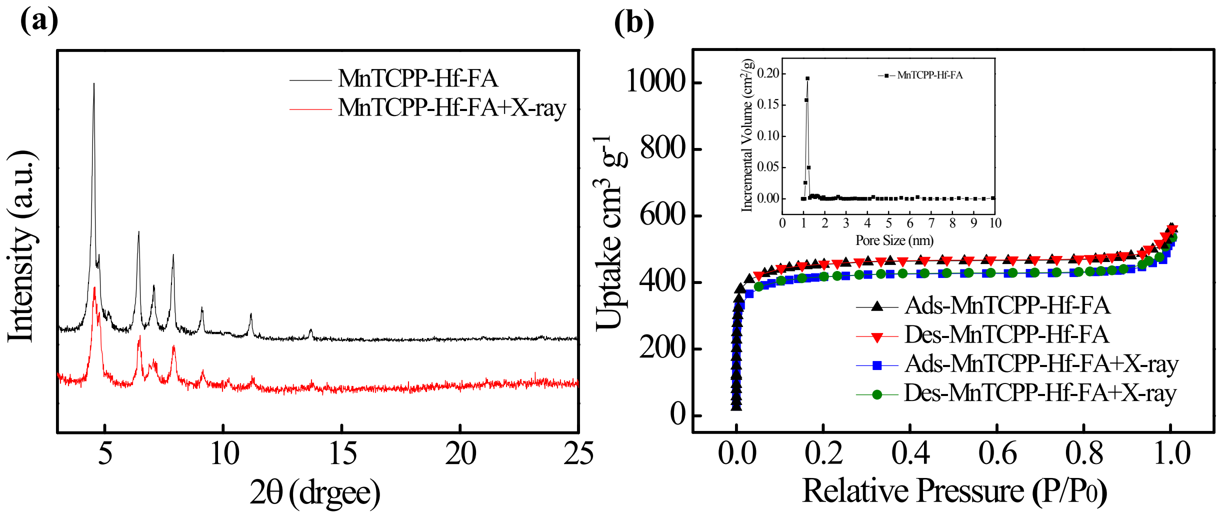

Figure S12. The radiation stability of MnTCPP-Hf-FA nanoparticles under 4 Gy X-ray irradiation evaluated using PXRD and N_2 isotherms. (a) PXRD of MnTCPP-Hf-FA MOF and MnTCPP-Hf-FA MOF under 4 Gy X-ray irradiation; (b) N_2 isotherms of MnTCPP-Hf-FA MOF and MnTCPP-Hf-FA MOF under 4 Gy X-ray irradiation. Inset: Pore size distribution of MnTCPP-Hf-FA MOF using the data measured with N_2 at 77 K.

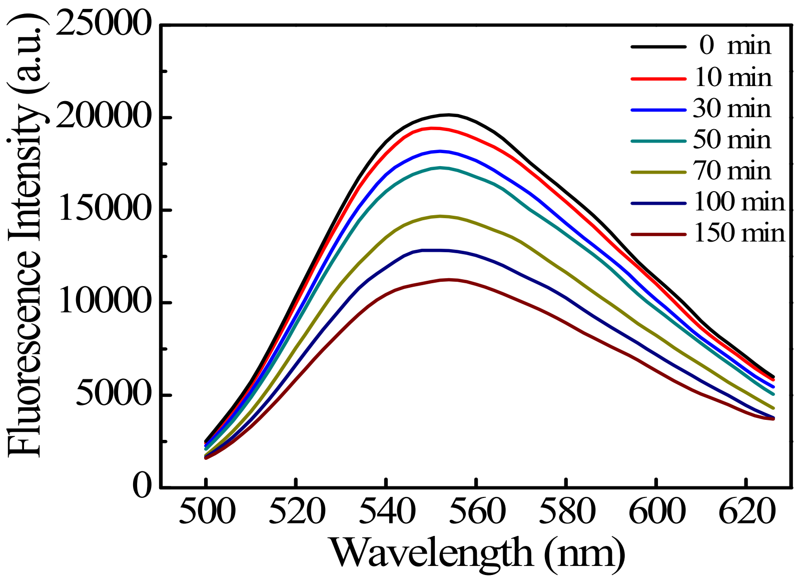

Fig. S13. Fluorescence intensity of MI-H_2O_2 for H_2O_2 detection in control group at different time.

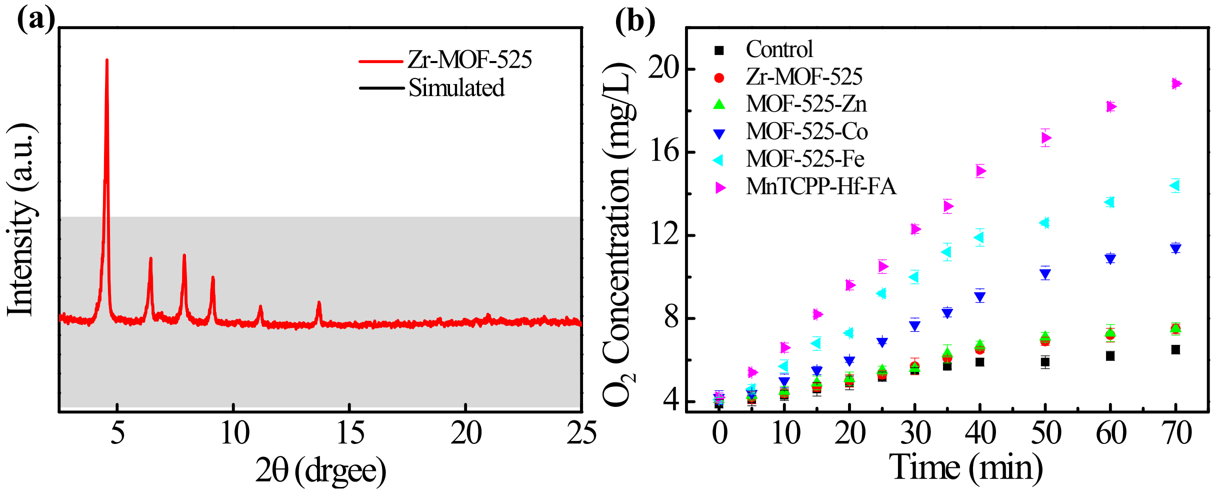

Figure S14. (a) The PXRD patterns of MOF-525 NPs (red line) and as-reported MOF-525 NPs (black line). (b) O_2 generation kinetics of H_2O_2 solution with different treatments.

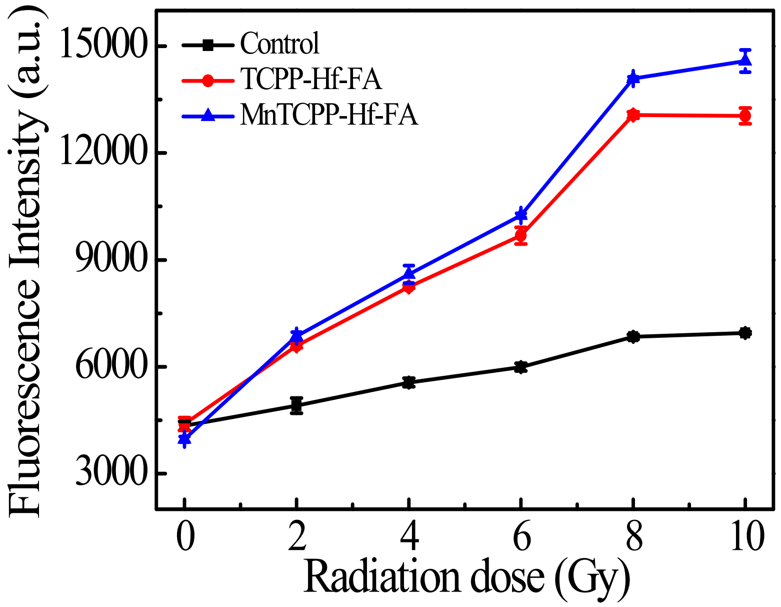

Fig. S15. Generation of ROS, as measured by the fluorescence intensity of SOSG with different doses of X-ray irradiation.

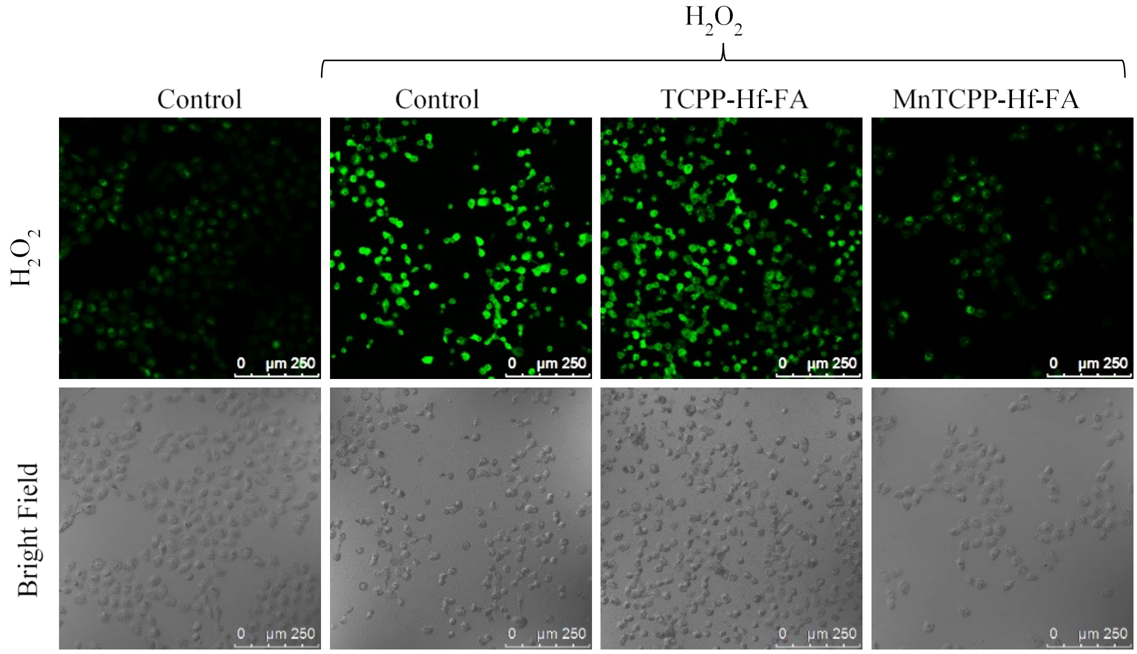

Fig. S16. CLSM images of H_2O_2 in cells after different treatments and labeled with Qcy7-H_2O_2. The H_2O_2 concentration was 150 μM.

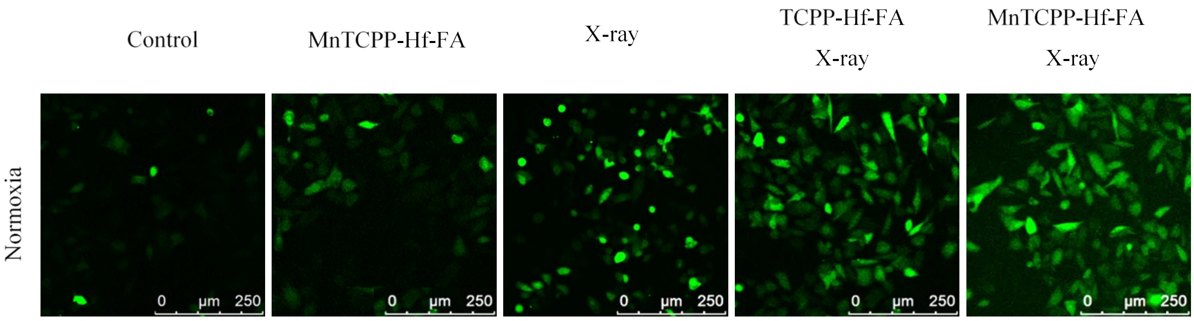

Fig. S17. The generation of ROS measured by CLSM after B16-F10 cells treated differently under normoxic condition after labelled with DCFH-DA (λ_{ex} = 488 nm, λ_{em} = 500 - 550 nm).

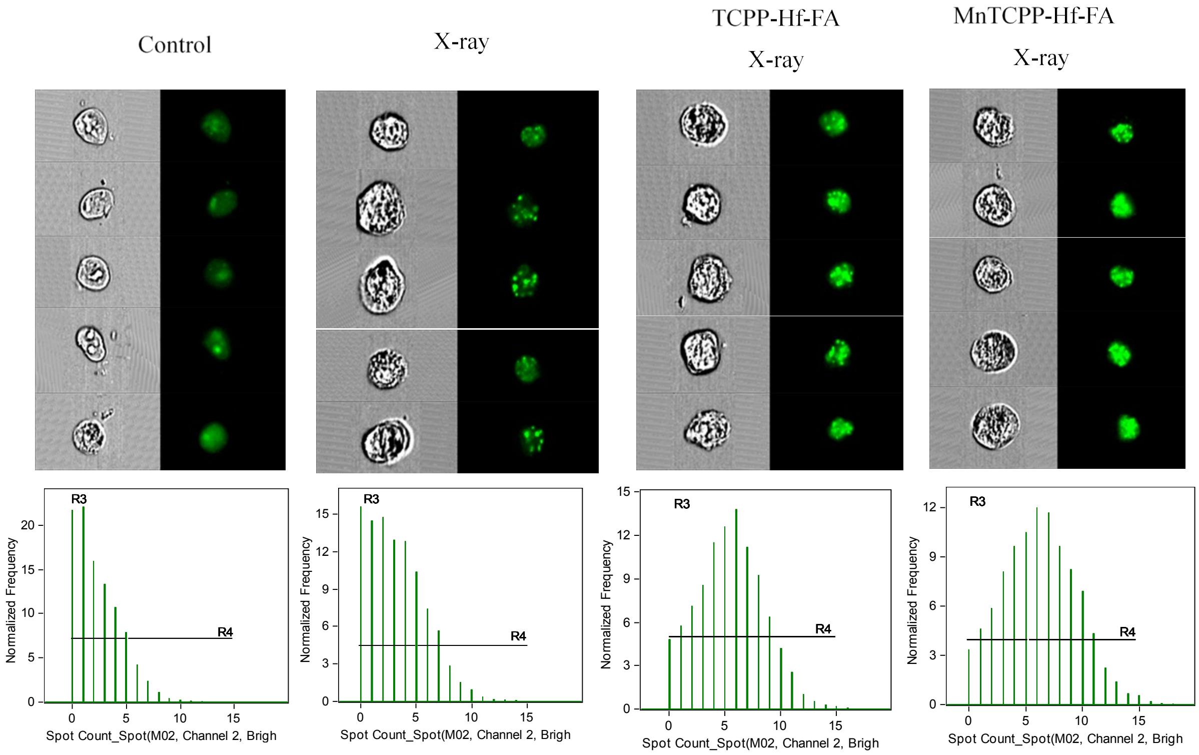

Fig. S18. Verification of DNA double strand breaks in B16-F10 cells *via* γ-H2AX immunofluorescence staining using imaging flow cytometry in normoxic condition.

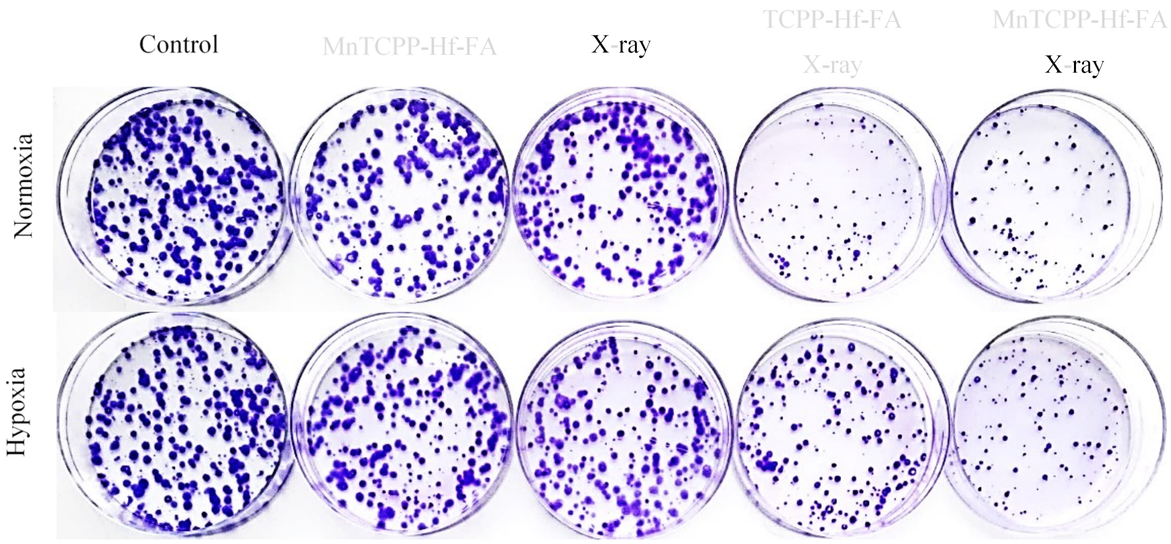

Fig. S19. Representative photographs of the colony formation assay under normoxic/hypoxic conditions.

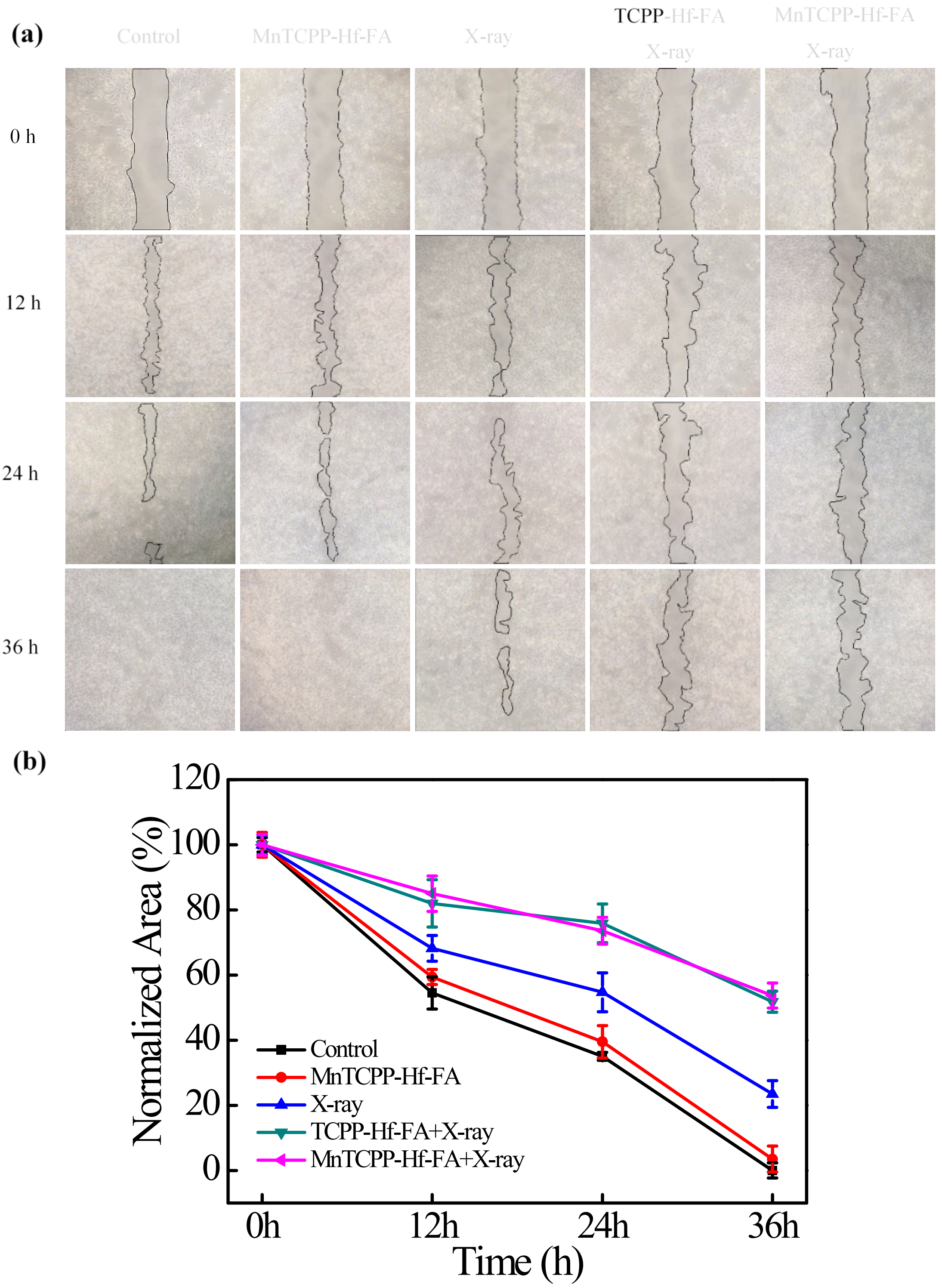

Fig. S20. (a) Representative photographs of cell migration under normoxic condition. Wound-healing assay of cells was treated differently and then incubated for different time after wounding. The black lines indicate the wound edge; (b) Corresponding quantitative data of the scratch area.

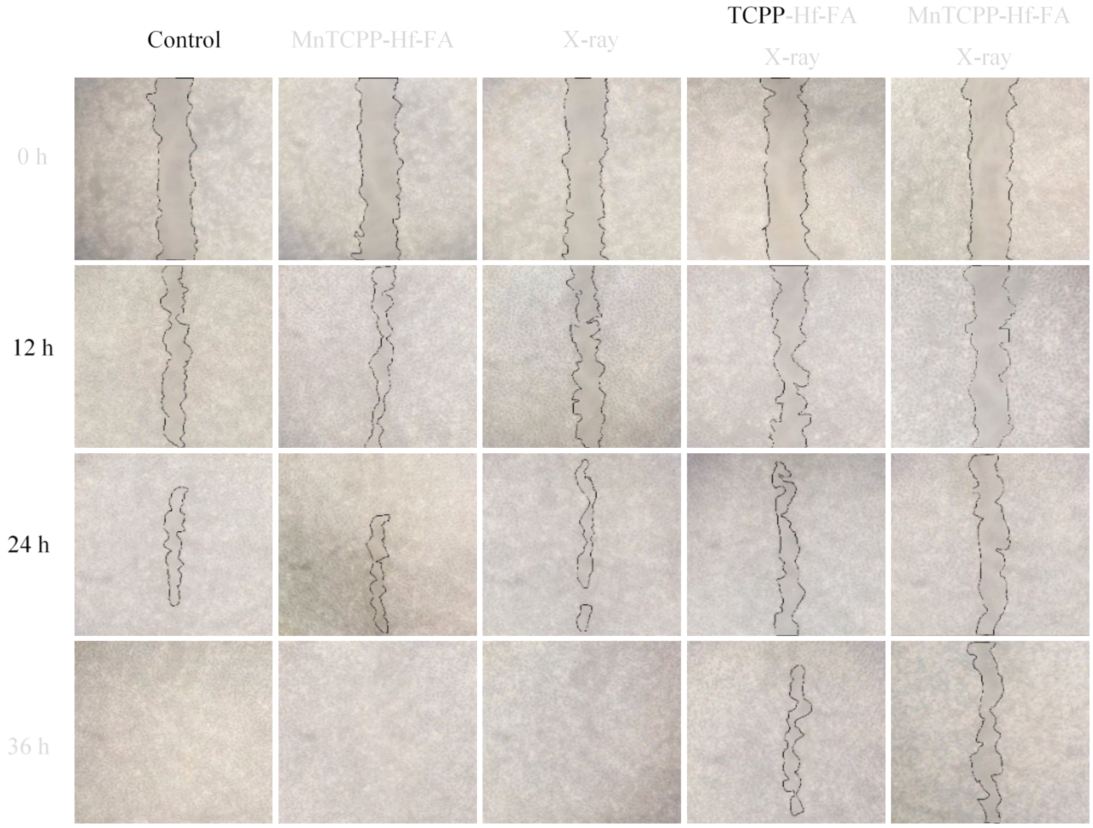

Fig. S21. Representative photographs of cell migration under hypoxic condition. Wound-healing assay of cells was treated differently and then incubated for different time after wounding. The black lines indicate the wound edge.

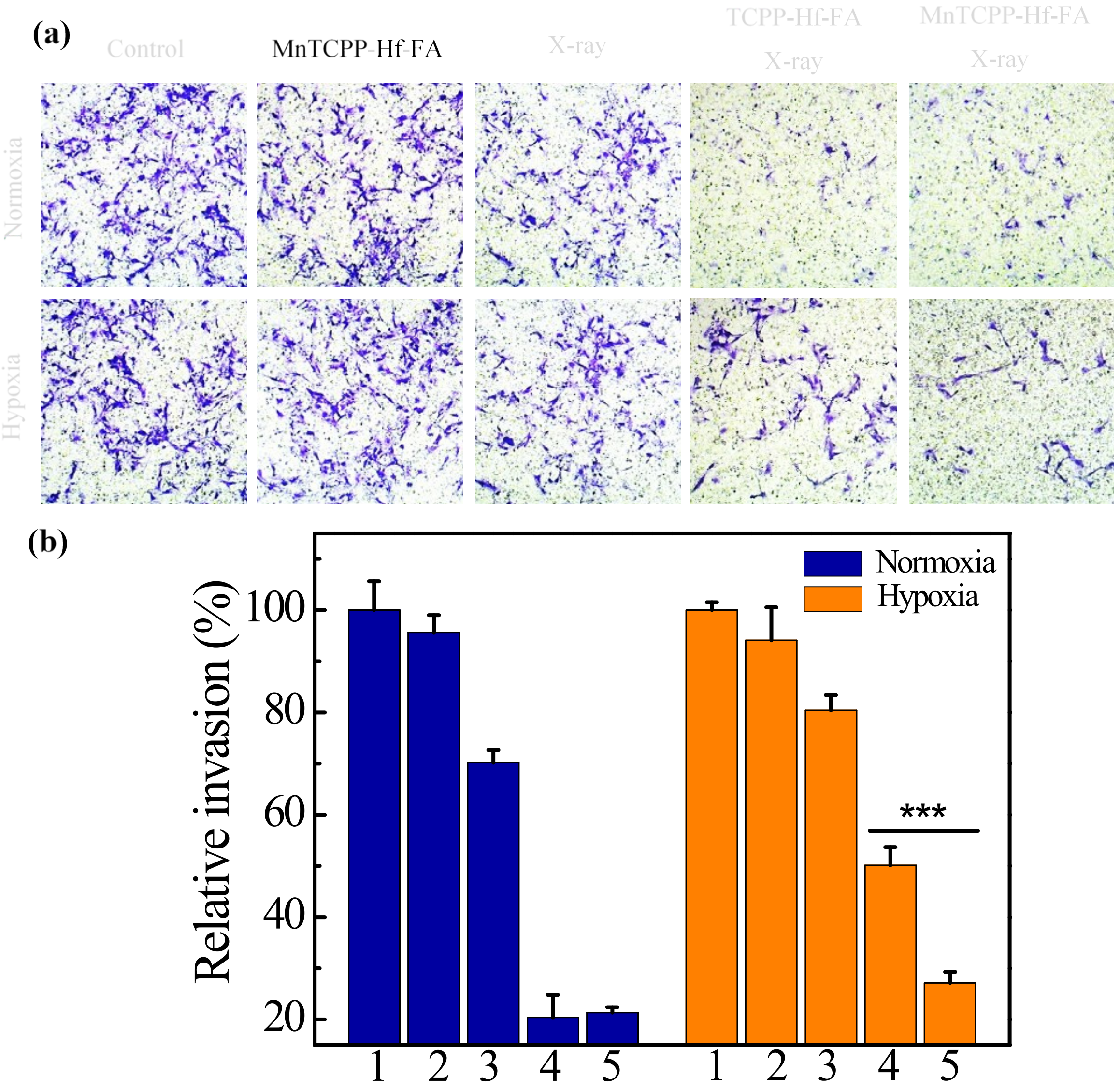

Fig. S22. Representative photographs of cell invasion under normoxic/hypoxic condition. The invasive cells were treated differently and then photographed (a). Corresponding quantitative data of invasive cells with different treatments (b) (student's t-test, ***p< 0.001).

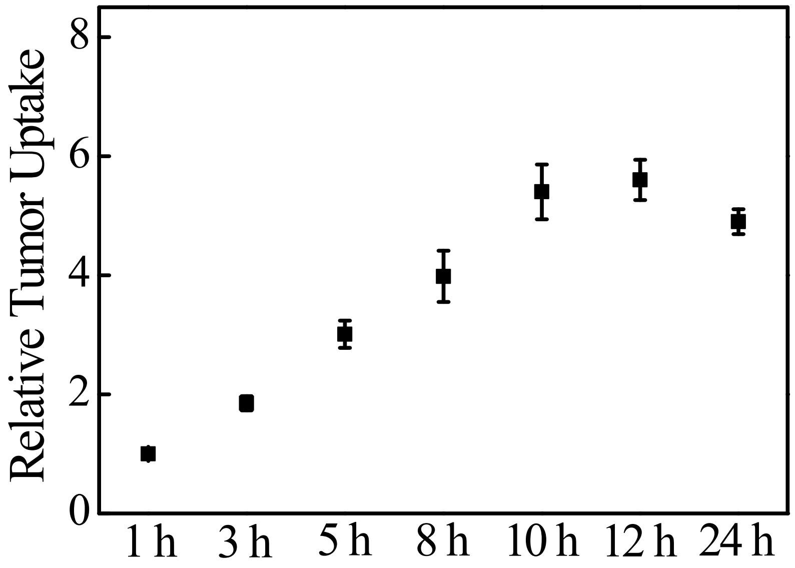

Fig. S23. The change in relative tumor uptake ($ID \bullet g_h^{-1}$/ $ID \bullet g_l^{-1}$) with time (1, 3, 5, 8, 10, 12, 24 h) after post-injection with MnTCPP-Hf-FA MOF. At the specific timepoints, the mice were sacrificed and the tumor tissue were harvested for the analysis of Hf element via ICP-AES.

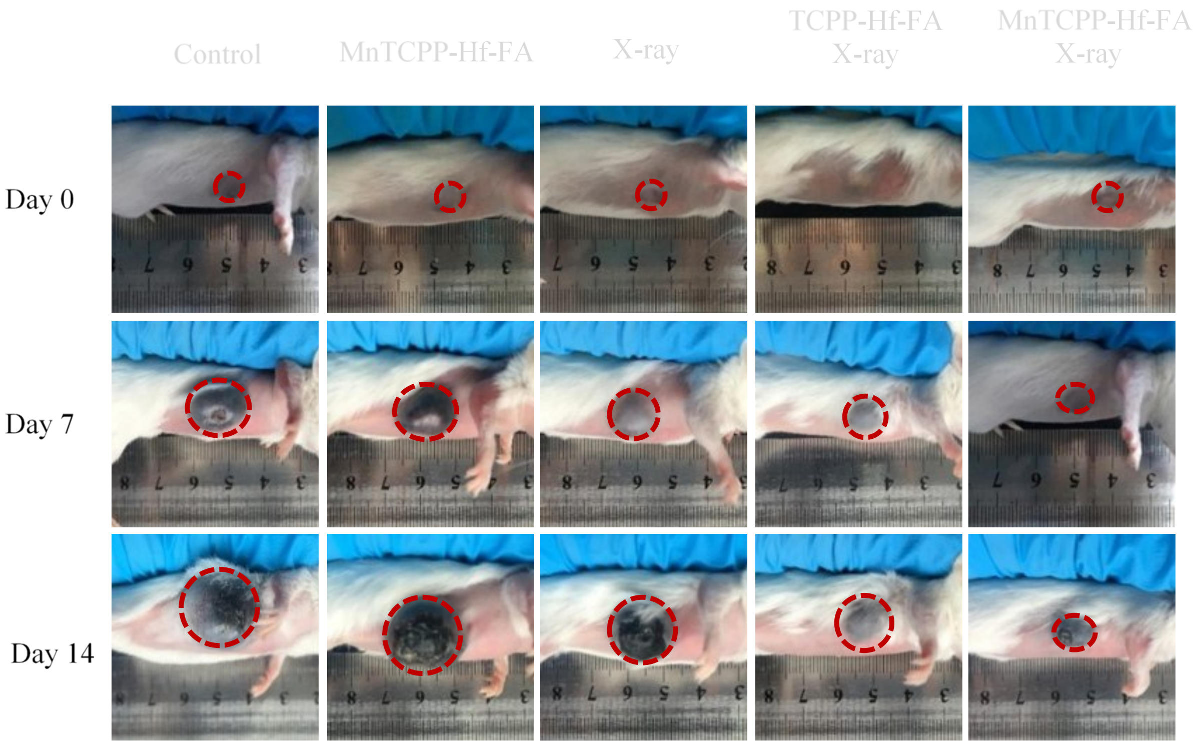

Fig. S24. In vivo antitumor study in a mouse model. Photographs of the mice before (0 day) and after (7 days and 14 days) the various indicated treatments

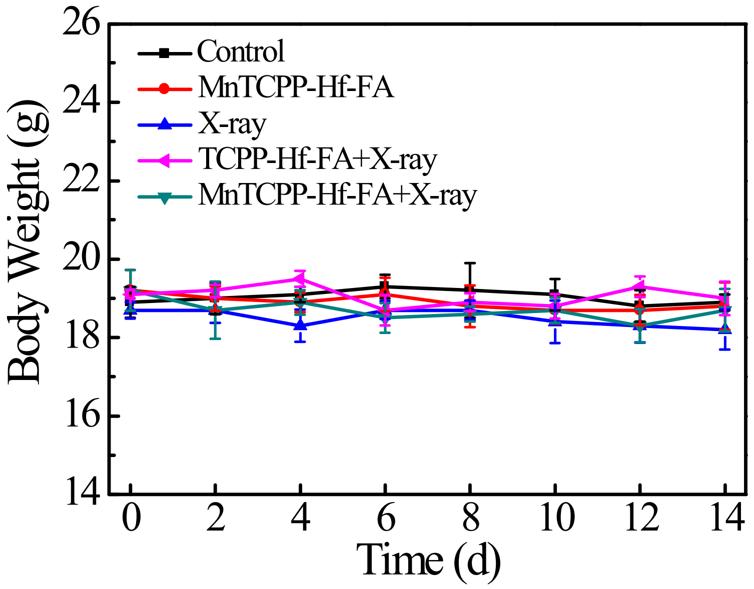

Fig. S25. Body weight changes of mice in various groups after receiving different treatments.

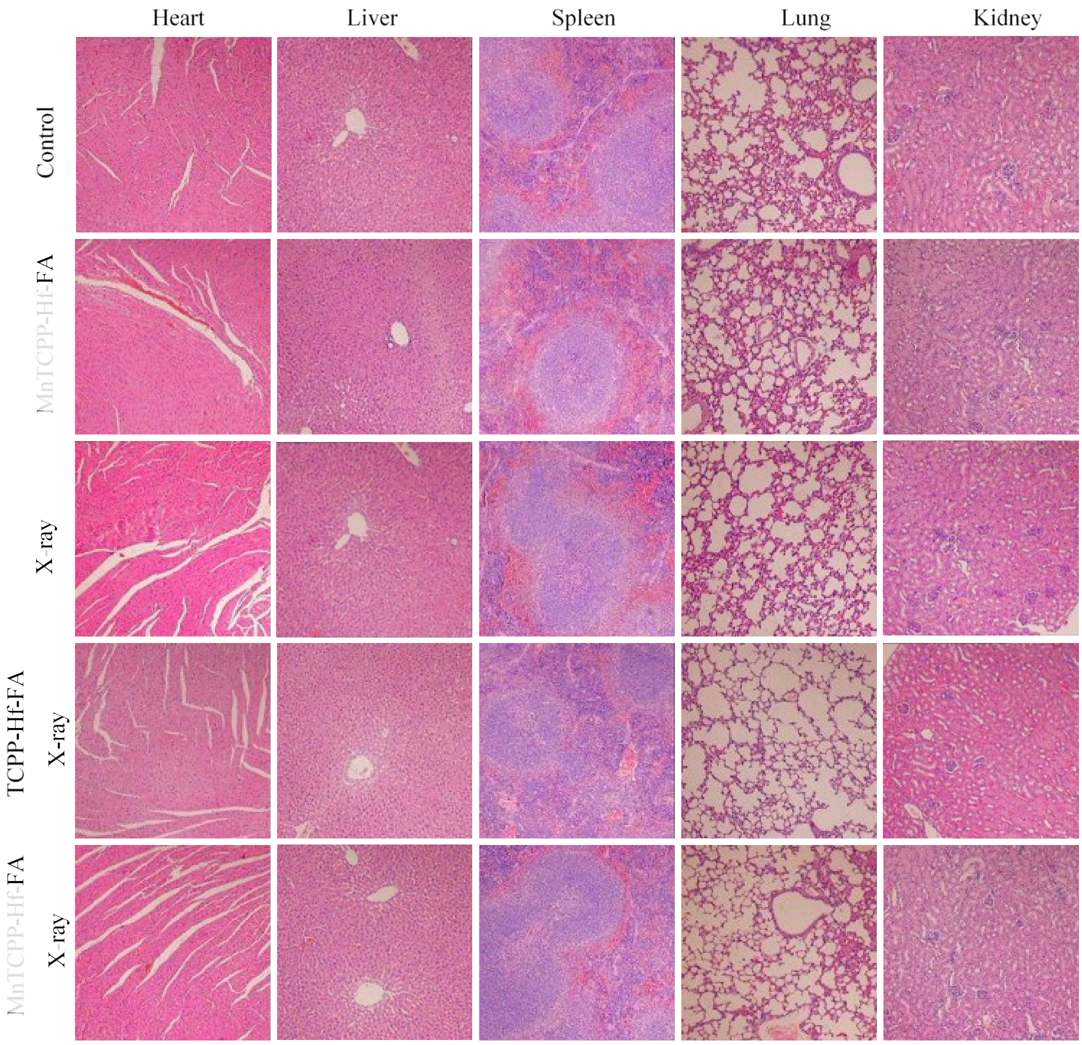

Fig. S26. H&E stained images of tissue slices of major organs (heart, liver, spleen, lung, kidney) at treatment 7 days obtained from different mice.

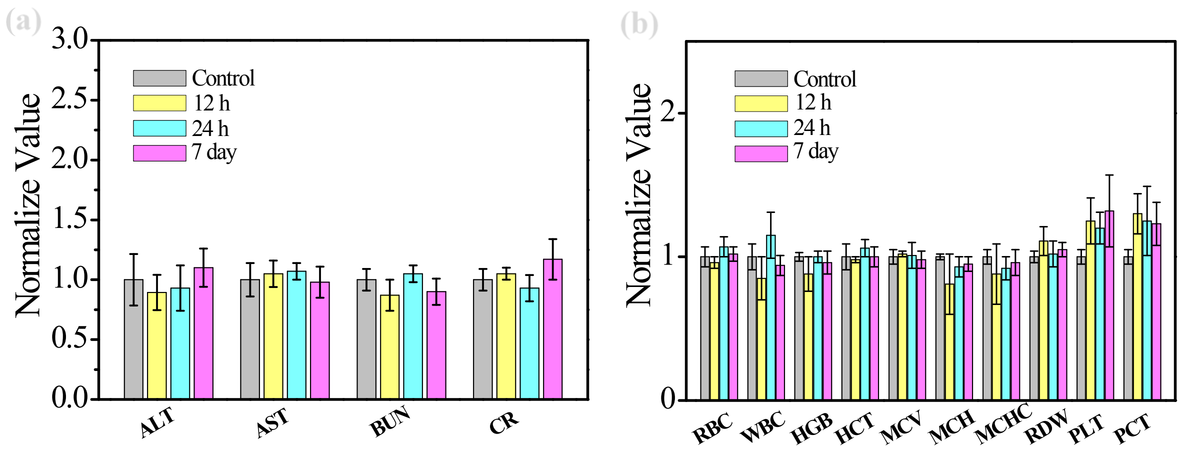

Fig. S27. (a) Blood biochemistry and (b) hematology data of mice at 12 h, 24 h and 7 days after intravenous injection with MnTCPP-Hf-FA MOF and intravenous injection with saline as control.

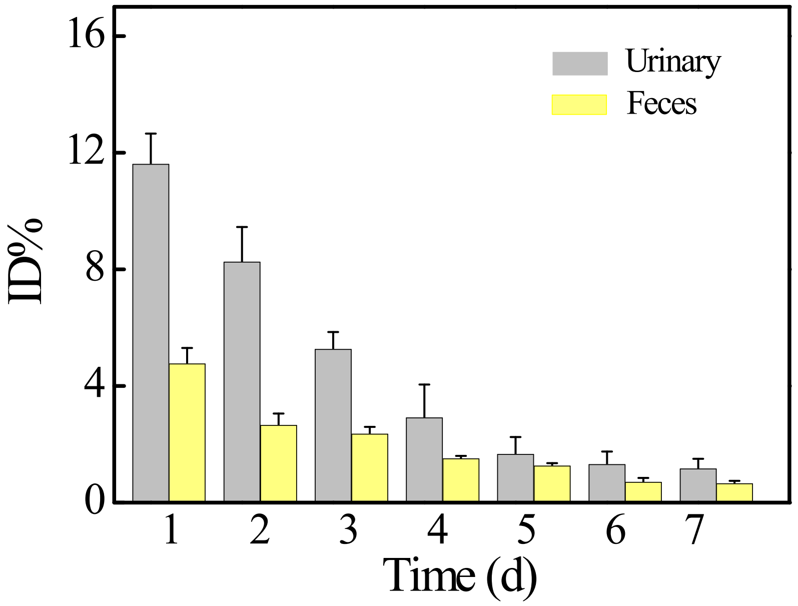

Figure S28. The detected Hf^{4+} mass in feces and urine at different time points post i.v. injection of MnTCPP-Hf-FA MOF.

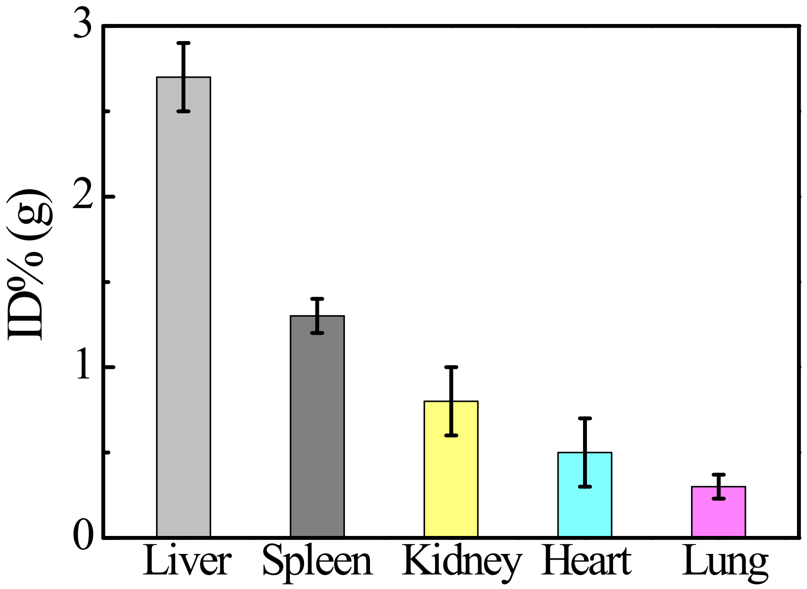

Fig. S29. At 7 days post-treatment, the mice were sacrificed and the organs were harvested for the analysis of Hf element *via* ICP-AES.

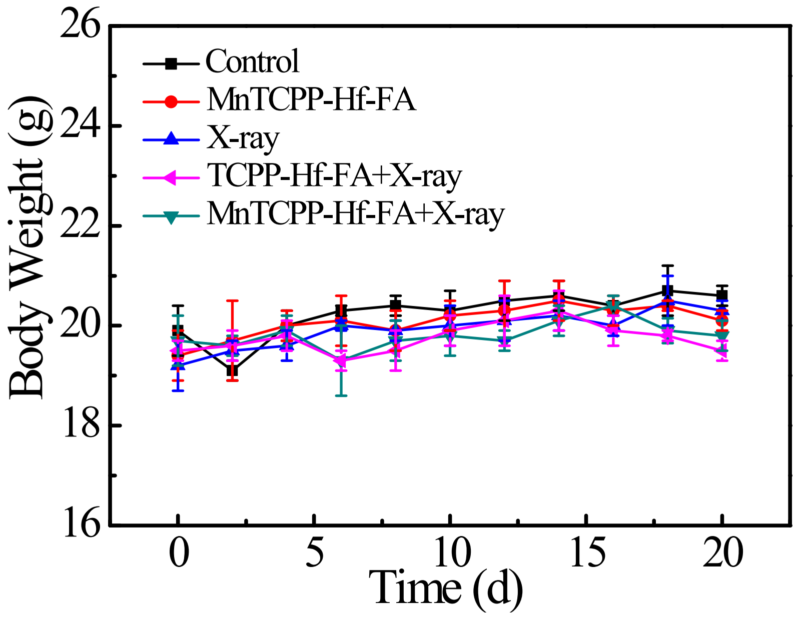

Fig. S30. Mice body weight curves of different groups of tumor-bearing mice. They were measured at 2 days interval for 20 days.
